# Towards peptide vaccines against Zika virus: Immunoinformatics combined with molecular dynamics simulations to predict antigenic epitopes of Zika viral proteins

**DOI:** 10.1038/srep37313

**Published:** 2016-12-09

**Authors:** Muhammad Usman Mirza, Shazia Rafique, Amjad Ali, Mobeen Munir, Nazia Ikram, Abdul Manan, Outi M. H. Salo-Ahen, Muhammad Idrees

**Affiliations:** 1 Center for Research in Molecular Medicine (CRiMM), The University of Lahore, Pakistan; 2 Centre for Applied Molecular Biology, University of the Punjab, Lahore, Pakistan; 3 Division of Science and Technology, University of Education Lahore, Pakistan; 4 Institute of Molecular Biology and Biotechnology (IMBB), The University of Lahore, Pakistan; 5 Structural Bioinformatics Laboratory, Faculty of Science and Engineering, Biochemistry, Åbo Akademi University, Turku, Finland; 6 Pharmaceutical Sciences Laboratory, Faculty of Science and Engineering, Pharmacy, Åbo Akademi University, Turku, Finland; 7 Vice Chancellor Hazara University, Mansehra, Pakistan

## Abstract

The recent outbreak of Zika virus (ZIKV) infection in Brazil has developed to a global health concern due to its likely association with birth defects (primary microcephaly) and neurological complications. Consequently, there is an urgent need to develop a vaccine to prevent or a medicine to treat the infection. In this study, immunoinformatics approach was employed to predict antigenic epitopes of Zika viral proteins to aid in development of a peptide vaccine against ZIKV. Both linear and conformational B-cell epitopes as well as cytotoxic T-lymphocyte (CTL) epitopes were predicted for ZIKV Envelope (E), NS3 and NS5 proteins. We further investigated the binding interactions of altogether 15 antigenic CTL epitopes with three class I major histocompatibility complex (MHC I) proteins after docking the peptides to the binding groove of the MHC I proteins. The stability of the resulting peptide-MHC I complexes was further studied by molecular dynamics simulations. The simulation results highlight the limits of rigid-body docking methods. Some of the antigenic epitopes predicted and analyzed in this work might present a preliminary set of peptides for future vaccine development against ZIKV.

Zika virus (ZIKV) is a positive-sense single-stranded RNA virus that belongs to the family of Flaviviridae. Its 10.7 kb genome encodes a single polyprotein. Viral and host proteases cleave this polyprotein into seven non-structural (NS1, NS2A, NS2B, NS3, NS4A, NS4B, and NS5) and three structural proteins (capsid – C, premembrane/membrane protein – prM/M, and the envelope – E)^1^.

Since its outbreak in 2015 in Brazil, ZIKV infection has spread rapidly in the tropical Americas. Its emergence dates seventy years back in Uganda’s Zika forest, when ZIKV was known to cause mild feverish infection^2^. Changes in the medical signs and symptoms of ZIKV were noted subsequent to its emergence in French Polynesia and an entrance in Latin America. Today Zika viral infection is seen to be followed by Guillian-Barré Syndrome (GBS)^3^ and adult ZIKV infection is linked with other sensory neurologic complications as well^4^. Due to Zika viral infection primary microcephaly and spontaneous abortion during first two pregnancy trimesters has been widely noted^5^,^6^. Ocular lesions in surviving infants have been noted^7^,^8^. Zika fetal disorder accounts for the aforementioned complexities along with the neonatal neurological impairment during pregnancy. GBS has been observed to sporadically follow dengue viral infection^9^,^10^ but is apparently more severe in the case of ZIKV due to the incidence of teratogenic lesions^11^. ZIKV has been identified by PCR in fetuses, and Zika virus particles have been detected by electron microscopy in the fetal central nervous system in high concentrations^5^. The fact that GBS and Zika fetal syndrome occur simultaneously, suggests that these syndromes might share a common etiology such as an autoimmune response that targets neurologic functions, raising the possibility of epitope mimicry^12^,^13^. Furthermore, high viral loads of ZIKV identified in the semen of infected patients, have indicated the possibility of its transmission by sexual means^14^. Lack of therapeutics or approved vaccines against ZIKV, till date, makes it difficult to control and prevent the infection. As a matter of fact, considerable endeavors are in focus to enhance comprehension of the vulnerability and pathogenesis of ZIKV. Envelope protein (E), NS3 and NS5 have been identified as potential targets for therapeutics and vaccines development. These targets have been recognized to have a major role in viral entry into the cell and in viral replication^15^,^16^. The risk associated with autoimmune responses to potential epitope mimics must be addressed in the development of vaccines and therapeutics for Zika virus infections^13^. Availability of genomic and immunological data and computer algorithms has brought about a more efficient process in vaccine development that allows for identification of possible epitopes which can assist the production of effective subunit vaccines^17^,^18^,^19^.

One of the indispensable and significant steps of vaccine development is the recognition of exceedingly competent B-cell linear (continuous) or conformational (discontinuous) and Cytotoxic T-lymphocyte (CTL) epitopes. It has been reported that approximately >90% of B-cell epitopes are discontinuous^20^,^21^. The target vaccine has been reported to elicit competent immune responses where T cells act as mediators. The progress in vaccine-designing research recently, has been facilitated by the development of refined assays designed to measure the T-cell responses against various vaccine candidates^22^. In order to explore T-cell epitopes in peptide sequences, aforementioned reports have accelerated research leading to the development of immunoinformatics methods^23^. With the help of immunoinformatics approach, we tried to make it possible to precisely narrow down potential B-cell and T-cell epitopes that can be characterized as effective vaccine candidates. Identifying which peptide of the virulent bacterial pathogen’s proteome binds to the major histocompatibility complex (MHC) molecules is considered as the first step to vaccine design. T-cell immunogenicity is correlated to the strength with which epitopes bind to MHC molecule^24^. With the help of appropriate molecular modeling tools, peptide-MHC complexes were modeled and their post-docking interaction studies helped further towards the selection of potential candidates for the development of peptide vaccines.

## Results

For designing peptide vaccines, this study aimed at identifying potential B cell and T cell epitopes with the help of an immunoinformatics approach. The identification of new vaccines by *in silico* approach can be carried out through the analysis of pathogenomics on a genome-wide scale^25^. There exist several limitations with respect to the conventional experimental approaches. With the help of immuno informatics, analysis of the complete spectrum of potential antigens is possible. Furthermore, the non-feasibility of pathogen culturing and the problems in the expression of antigens *in vitro* can also be circumvented^26^. and several *in silico* vaccine candidates reported by immune research groups have been known to produce promising preclinical results^26^.

In the current study, potential B-cell epitopes (continuous and discontinuous) and T-cell epitopes have been identified for the designing of peptide vaccines against ZIKV E, NS3, and NS5 proteins. With the help of immunoinformatics approach as well as docking tools for predicting peptide-protein complexes, the analysis was performed because these tools are very helpful for the identification of new vaccines^25^. Numerous researchers have documented *in silico* approach to design vaccines with promising clinical trials^27^. *In silico* process helps in reducing the number of *in vitro* experiments, hence, prediction of B-cell epitopes and CTL epitopes by various immunoinformatics approaches serves as an essential tool in vaccine design^28^,^29^.

### Prediction of Antigenic B-Cell Epitopes

Based upon the physicochemical characteristics of amino acids that have been observed in experimentally determined antigenic epitopes, Kolaskar and Tongaonkar’s method^30^ was employed to predict antigenic epitopes of a given sequence. It has been reported that this method provides 75% experimental accuracy^30^. This procedure predicted that the ZIKV E protein sequence of 403 amino acids has 21 antigenic peptides that fall in the range of 7–18 amino acids with 6 heptapeptides (Table 1). Similarly, it was predicted that the sequence of NS3 of 151 amino acids contains 6 antigenic peptides. The length of the antigenic peptides was 7–21 amino acids with 2 hendeca-peptides for NS3 (Table 2). Moreover, the sequence of NS5 of 641 amino acids has 22 antigenic peptides. The length of the antigenic peptides was 6–25 with 6 nonapeptides for NS5 (Table 3). Furthermore, Kolaskar and Tongaonkar’s method also predicted the maximum residual score for each amino acid in E, NS3, and NS5. 252 out of 403 amino acids of ZIKV E protein has a residual score greater than 1.000. Leucine at the 258^th^ position, found in the antigenic peptide from position 252 to 260 (RQTVVVLGS), was identified as having the maximum residual score of 1.184.

Likewise, 100 out of 151 amino acids of ZIKV NS3 protein were predicted to have a residual score >1.000. Leucine at the 81^st^ position, in the antigenic peptide from position 76 to 86 (SEVQLLAVPPG), was identified as having the maximum residual score of 1.201. It should be noted that the leucines at positions 80 and 81 from this ZIKV NS3 antigenic site were also predicted as comprising a CTL epitope, thus significantly enhancing the scope of amino acid leucine at positions 80 and 81 to be treated as potential peptide vaccine candidates. In ZIKV NS5,322 out of 641 amino acids were predicted to have a residual score >1.000 and serine at 533^rd^ position of the antigenic peptide from position 528 to 539 (NAICSSVPVDWV), had the maximum residual score of 1.203.

Graphical representation of the predicted peptides of B-cell E, NS3 and NS5 proteins on the basis of their sequence position (along the x-axis) and antigenic propensity (along the y-axis) are shown in Fig. S1 (Supporting Information). The variation of antigenic propensity is associated with sequence length. The minimum antigenic propensity score for the ZIKV E protein was 0.861 while the maximum score was 1.186 (A). Moreover, the average antigenic propensity score came out to be 1.026 (A). On the other hand, minimum and maximum (B,C) value for NS3 and NS5 (0.911 and 0.851) and (1.202 and1.203) was observed respectively. The average antigenic propensity score for NS3 and NS5 came out to be 1.035 (B) and 1.008 (C) respectively.

### Surface accessibility for E protein, NS3, and NS5

The surface probability of a hexapeptide more than 1.0 (threshold) predicts that the sequence has an increased probability to be found on the surface^31^. The graphical representation of the predicted peptides for E protein, NS3 and NS5 depending upon the sequence position (along the x-axis) and surface probability (along the y-axis) is shown in Fig. S2. The minimum surface probability score calculated by the program was 0.067 for the E protein from amino acid position 387 to 392 (387IVIGVG392), while the maximum surface probability score calculated was 5.725 for the E protein from amino acid position 159 to 164 with the sequence of hexapeptide 159ETDENR164, where E159 is a surface residue (one with a greater than 20 Å^2^ of water-accessible surface).

Moreover, the minimum surface probability score calculated by the program was 0.123 and 0.067 for NS3 and NS5 from the amino acid position 19 to 24 and 314 to 319, respectively. The maximum surface probability score calculated was 4.935 and 9.228 for NS3 and NS5 from the amino acid position 100 to 105 and 207 to 212 with the sequences of hexapeptides 100KTKDGD105 and 207KREKKQ212, where K100 and K207 are surface residues as shown in Fig. S2.

### Surface flexibility for E protein, NS3, and NS5

The vibrational motion of atoms within a structure indicated by temperature or B factors is calculated by Karplus and Schulz flexibility method^32^. In well-ordered, that is, organized structure, the atoms have a low B-factor value; on the other hand, the higher the B factor^32^. The graphical representation of the surface flexibility results for E, NS3, and NS5 proteins are shown in the Fig. S3. The minimum flexibility score 0.894 was predicted by the program showing a more ordered structure with a sequence of heptapeptide 137YRIMLSV143 while the maximum score was 1.112 (367ESTENSK373 amino acid sequence) for E protein. On the other hand, the minimum flexibility score for NS3 and NS5 were predicted to be 0.887 and 0.839 respectively, showing a more ordered structure with a sequence of heptapeptides 29FHTMWHV35 and 223AIWYMWL229 respectively, while the maximum score was 1.117 and 1.125 (115AGTSGSP121 and 250RENSGGG256 amino acid sequences) respectively.

### Parker Hydrophilicity Prediction for E protein, NS3, and NS5

Based upon the peptide retention times during HPLC on reversed phase column, the Parker hydrophilicity scale method is employed for the prediction of hydrophilicity of peptides^33^. The depicted surface hydrophilic regions are associated with the known antigenic sites in immunological studies^33^.

The graphical representation of the predicted peptides for E protein, NS3 and NS5 on the basis of the sequence position (along the x-axis) and hydrophilicity (along the y-axis) is shown in Fig. S4. The minimum hydrophilicity score calculated by the software was −3.171 for E protein from amino acid position 217 to 223. According to the Parker Surface Hydrophilicity Prediction result is 217WFHDIPL223, the sequence of a heptapeptide. The maximum hydrophilicity score calculated was 7.057 for E protein from amino acid position 83 to 89 with the sequence of a heptapeptide 83DKQSDTQ89.

Moreover, the minimum hydrophilicity score calculated by the program was −2.529 and −5.886 for NS3 and NS5 from amino acid position 28 to 34 as well as 29 to 35 (NS3) and 223 to 229 (NS5), respectively. According to the Parker Surface Hydrophilicity Prediction result are 28VFHTMWH34 as well as 29FHTMWHV35 and 223AIWYMWL229 sequences of heptapeptides. The maximum hydrophilicity score calculated was 4.914 and 6.300 for NS3 and NS5 from amino acid position 73 to 79 and 347 to 353 as well as 348 to 354 with the sequences of heptapeptides 73DGHSEVQ79 and 347QDQRGSG353 as well as 348DQRGSGQ354, respectively.

### CTL epitope prediction

Cytotoxic T-lymphocyte (CTL) epitopes were investigated from the ZIKV. The infected cell with antigen-presentation activates T-cell to become effector cell to kill any infected cell. Self-destruction or cell death is observed after the CTLs attack on infected cells. The peptide fragment of the pathogen and the MHC molecule bind together and appear on the cell surface of infected cells. The peptide-protein complex is recognized by CTLs, and, as a result, infected cells are killed. Peptide fragment (antigen) processing, as well as its presentation to the T-cell, is accomplished by various steps. Peptides are processed in the cytosol by the proteasome and later transported to the endoplasmic reticulum (ER) where MHC is synthesized. Here, transporter associated with antigen processing (TAP) moves the peptide into the MHC I molecule. After that peptide-MHC I complex is transported to the cell surface. A diverse range of peptides is bound to each allelic form of MHC I protein. The MHC molecule has the ability to bind with peptides tightly as the pathogens try to mutate the epitope of the MHC molecule. Hence, MHC molecule exhibits high binding affinity with a variety of peptides^34^.

In the scope of *in silico* vaccine designing, T-cell epitope prediction is rendered as a milestone^28^. CTL epitopes are proved to be potential candidates for peptide vaccine design for various diseases. By using *in silico* epitope prediction methods, numberless research groups have reported excellent results. In the development of different vaccines against various infectious diseases, including autoimmune disease and cancer, these research groups have been found to have played a key role^35^.

CTL epitope prediction is an important *in silico* tool in the vaccine designas it reduces the need for *in vitro* experiments. NetCTL 1.2 server^36^ was employed for the CTL epitope prediction. Peptide sequences from ZIKV-E, NS3 and NS5 were predicted as CTL epitopes on the basis of their specific MHC binding affinity, proteasomal C-terminal cleavage, TAP transport affinity and potential MHC ligands were identified, whose prediction scores were >0.75000 threshold. Ten peptide sequences from ZIKV-E were predicted as CTL epitopes whose prediction score were >0.75000 (Table 4). For ZIKV-NS3, five peptide sequences were predicted as CTL epitopes with predicted score above threshold (Table 5). As mentioned above, the leucine residues at positions 80 and 81 were also predicted as antigenic sites. Hence, 75HSEVQLLAV83, one of the CTL epitopes predicted from ZIKV-NS3, can be considered a potential vaccine candidate. Correspondingly, fifteen peptide sequences from ZIKV-NS5 were predicted as CTL epitopes whose prediction score were >0.75000 (Table 6).

### Homology modeling and structure validation

To perform structure-based epitope prediction of ZIKV proteins (E, NS3 and NS5), homology modeling of proteins E, NS3 and NS5 was performed (Fig. S5). It was observed that the 3D crystal structures of dengue virus type 1 and type 3 envelope proteins (3G7T chain A and 1UZG chain A), West Nile virus NS2b-NS3 protease in complex with 3, 4-dichlorophenylacetyl-Lys-Lys-GCMA (2YOL chain A) and RNA-dependent RNA polymerase domain from the West Nile virus (2HFZ chain A) were found to be the best hits based on E-value, query coverage and identity. Hence, these were considered the best templates to perform homology modeling (Table 7).

HHpred server^37^ identified 57% maximum identity of glycoprotein E for ZIKV (403 amino acids) with the Protein Data Bank (PDB) structures: 3G7T chain A (crystal structure of dengue virus type 1 envelope protein) and 1UZG chain A (crystal structure of dengue virus type 3 envelope protein). The program employed PDB codes (3G7T chain A and 1UZG chain A) as a template and built the homology model with “100%” confidence. Greater than 90% confidence reflects that core model is very much precise and correct deviating 2–4 Ǻ in rmsd from the native protein structure. Moreover, the percentage identity between the template and the query sequence being more than 30–40% reflects good accuracy in the model.

As far as NS3 and NS5 proteins are concerned, 70% (NS3) and 72% (NS5) maximum sequence identity for ZIKV were found with the PDB structures 2YOL chain A (crystal structure of West Nile virus NS2b-NS3 protease in complex with 3, 4-dichlorophenylacetyl-Lys-Lys-GCMA) and 2HFZ chain A (crystal structure of RNA-dependent RNA polymerase domain from West Nile virus). Stereochemical quality of the models was also comparable with the template structures (see Methods).

### Structure-based Epitope Prediction for ZIKV E, NS3 and NS5 proteins

For the epitope prediction in 3D structures, ElliPro^38^ was employed. This web-based advanced program helps studying the correlation between solvent accessibility, flexibility, and antigenicity of a protein structure. Moreover, an important feature is that the predicted epitopes are differentiated on the basis of protein-antibody interactions. For E-protein of ZIKV three-discontinuous peptides with a score value of 0.7 or more were selected. The score (Protrusion Index, PI) reflects the percentage of protein atoms that extend beyond the molecular bulk and are responsible for antibody binding^38^.

**Figure 1:**
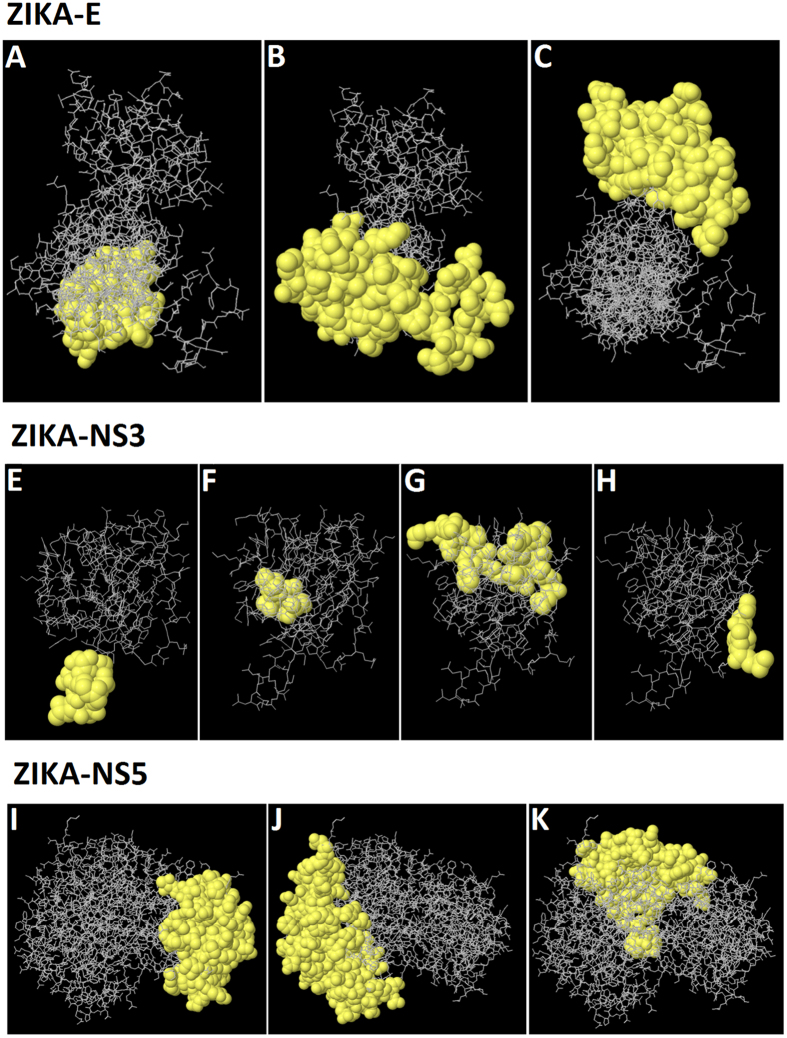
3D Representation of the predicted discontinuous epitopes (**A** to **C**) of Zika-E protein, (**E** to **H**) of Zika-NS3 protein and (**I** to **K**) of Zika-NS5 protein.

The highest probability for the E protein was computed as 74.4% (PI score: 0.744). Moreover, the highest probability for both NS3 and NS5 was computed as 87.1% and 73.6% (PI score: 0.871 and 0.736), respectively. Amino acid residues, the number of residues, sequence location as well as their scores are tabulated in the Tables 8, 9 and 10. The graphical representation of the discontinuous epitopes is displayed in Fig. 1.

### Molecular Docking of ZIKV-E protein with HLA-A0201

**Figure 2:**
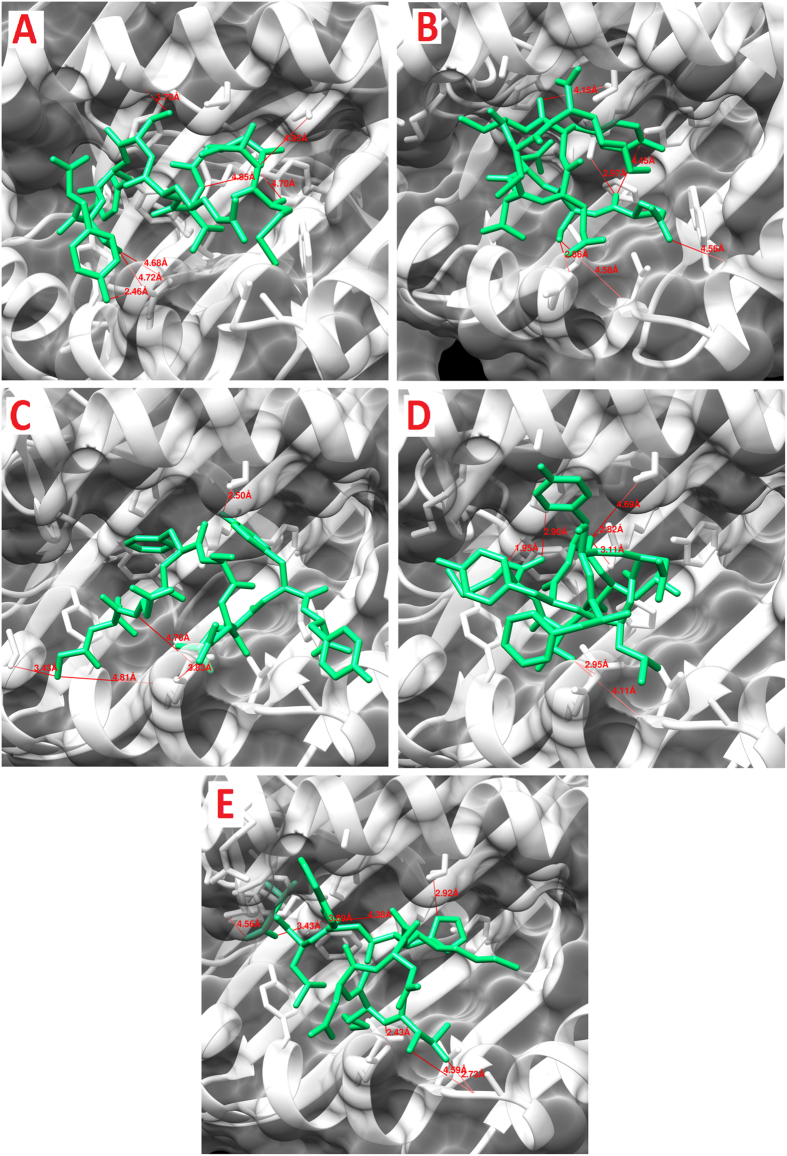
Molecular interaction analysis of predicted ZIKV E protein peptides docked to MHC-I HLA-A*02:01. (**A**) MAEVRSYCY; (**B**) QSDTQYVCK; (**C**) GLDFSDLYY; (**D**) FSDLYYLTM; (**E**) TMNNKHWLV.

Out of 10 epitopes predicted, five CTL epitopes predicted from ZIKV-E protein were docked to MHC class I HLA-A0201. All five CTL peptides exhibited strong binding affinities in terms of global energy and attractive van der Waals energy (vdW) ranging from −52.10 to −59.59 kcal/mol and −19.87 to−29.58 kcal/mol as tabulated in Table 11. Among the five peptides, post-docking analysis of three CTL predicted epitopes (MAEVRSYCY, FSDLYYLTM, and TMNNKHWLV) revealed good interactions with HLA-A0201 (Fig. 2). In MAEVRSYCY-MHC HLA-A0201 complex, three hydrogen bonds within a distance of 3.5 Å, suggested the stability to the docked complex. Significant hydrogen bond interactions were seen between the residues Cys8 and Tyr9 of the peptide and Asp77 of the MHC protein. It was further predicted that three amino acids (Ser6, Cys8, and Tyr9) were antigenic. Similarly, four hydrogen bonds were formed between FSDLYYLTM and HLA-A0201 (Table 11). Tyr6 formed hydrogen bond interactions through backbone oxygen atom with hydroxyl oxygen atom of Thr73 of the MHC protein. Tyr6 has been predicted to be antigenic. The docked complex of TMNNKHWLV-MHC HLA-A0201 showed five hydrogen bond formations within a distance of 3.5 Å. Overall stability of the docked complex seems to be well-preserved by the formation of hydrogen bonds. Residues Thr1, Met2, His6 and Val9 showed significant hydrogen bond interactions with Ala150, Gln155, Thr73, And Tyr99 of the MHC protein (Table 11). His6, Trp7, and Val9 of the peptide have been predicted to be antigenic. Molecular interactions of the specific peptides and their respective docked poses are represented in Fig. 2.

### Molecular Docking of ZIKV-NS3 protein with HLA-B2705

**Figure 3:**
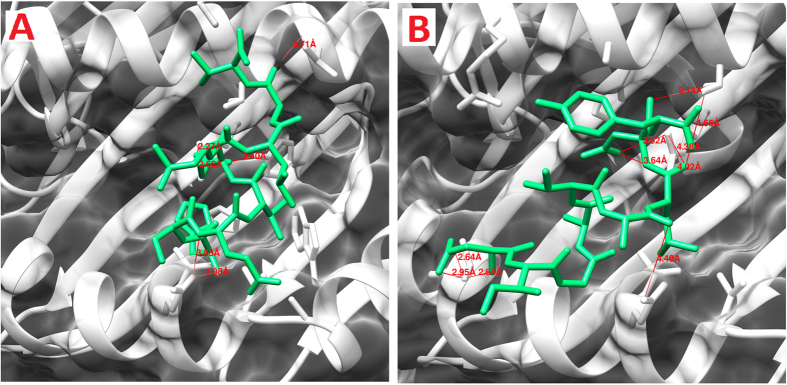
Molecular interaction analysis of predicted ZIKV NS3 peptides docked to MHC I HLA-B*27:05. (**A**) HSEVQLLAV; (**B**) DIGAVALDY.

Two CTL epitopes predicted from ZIKV-NS3 protein, out of five peptides, were docked to class I MHC-HLA-B2705. Docked complexes of HSEVQLLAV and DIGAVALDY peptides can be seen in Fig. 3. Four hydrogen bonds were formed between HSEVQLLAV peptide and the MHC protein with the interatomic distance within 3.5 Å. Residues Gln5 and His1 from the peptide and Tyr116, Arg97 and Gln155 were involved in hydrogen bonding (Table 12). On the other hand, the second peptide (DIGAVALDY) from NS3 protein formed four hydrogen bonds with MHC-HLA-B2705. Oxygen (OD1) of Asp8 from peptide formed one hydrogen bond with nitrogen (NH1) of Arg97 from the MHC protein. Moreover, Asp1 of the peptide formed three hydrogen bonds with Thr163 from the MHC protein. Backbone nitrogen and oxygen (OD1 and OD2) of Asp1 formed hydrogen bonds with the side chain oxygen (OG1) of Thr163. Residues Glu3, Gln5 and Ala8 from peptide HSEVQLLAV and Ala6 and Asp8 from DIGAVALDY were also predicted to be antigenic (Table 12). Molecular interactions of the specific peptides and their respective poses have been represented in Fig. 3.

### Molecular Docking of ZIKV-NS5 protein with HLA-C0801

**Figure 4:**
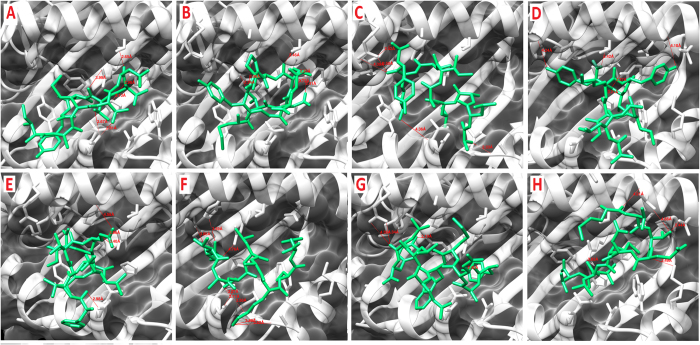
Molecular interaction analysis of predicted ZIKV NS5 peptides docked to MHC-I HLA-C*08:01. (**A**) IAMTDTTPY; (**B**) MTDTTPYGQ; (**C**) ALALAIIKY; (**D**) ALAIIKYTY; (**E**) FTNLVVQLI; (**F**) ETACLAKSY; (**G**) YAQMWQLLY; (**H**) MTTEDMLVV.

Eight CTL epitopes predicted from ZIKV-NS5 protein, out of 15 peptides, were docked against class I MHC-HLA-C0801. The docked complexes are shown in Fig. 4. The overall stability of the complex structures seems to be well preserved by the formation of hydrogen bonds (Table 13). It can be noted that common residues including Arg97, Tyr99, Tyr116 and Gln155 from MHC-HLA-C0801 were involved in the formation of hydrogen bonding with various CTL predicted peptides of ZIKV-NS5 protein. The docked structure of peptides IAMTDTTPY, MTDTTPYGQ, ALALAIIKY, ALAIIKYTY, FTNLVVQLI, ETACLAKSY, YAQMWQLLY and MTTEDMLVV showed promising global energy (−33.97 to −58.25 kcal/mol) and attractive vdW energy (−20.37 to −27.29 kcal/mol) as shown in Table 13.

Both nitrogens (NH1 and NH2) of Arg97 from the MHC-HLA-C0801 formed hydrogen bonds with the backbone oxygen atom of Gln7 of FTNLVVQLI. Likewise, Gln155 from MHC-HLA-C0801 formed several hydrogen bonds; hydrogen bond interactions were formed between oxygen (OE1) of Gln155 and OG1 of Thr4 and Thr7 of IAMTDTTPY, and as well as with sulphur (SG) of Cys4 (ETACLAKSY). The detailed depiction has been given in the Table 13 and illustrated in Fig. 4.

### Molecular dynamics simulations

The stability of the docked peptide-MHC I protein complexes was further investigated by performing molecular dynamics (MD) simulations of the complexes in an explicit water box at 300 K for a period of 5 nanoseconds. The potential energy of the simulation systems remained stable during the MD simulations (data not shown). Some of the docked complexes had steric clashes between the amino acids of the binding partners (see Tables 11, 12 and 13). However, during the energy minimization of the complexes, these clashes were removed and, on the other hand, new favorable interactions (e.g. hydrogen bonds) were formed (Tables 11, 12 and 13). A few peptides kept their initial conformation during the simulation, whereas others lost it partially (Supporting Information, Fig. S6-8; Table S1). Moreover, many peptides moved from their original docking position and formed new favorable interactions during the simulation (Tables 11, 12 and 13). The binding groove (‘F pocket’)^39^ also changed its size variably depending on the peptide that was inside the groove (Supporting Information, Table S1, Fig. S6–8).

## Discussion

The urgent need for preventive measures against a global threat of a ZIKV epidemic has awakened the researchers to investigate the pathogen. Especially cost-effective and fast methods such as immunoinformatics tools have been quickly harnessed by researchers in different countries to, for example, predict possible antigenic epitopes from ZIKV proteins (especially the E protein) for peptide vaccine development (see for example these very recent studies in refs 40, 41, 42, 43, 44, 45, 46, 47). In general, such *in silico* approaches help reduce the number of *in vitro* experimental assays and provide an essential tool in vaccine design^27^,^28^,^29^. In the current study, we have predicted potential epitopes not only for the E protein but also for the NS3 and NS5 proteins. Some of the recent studies have also employed different docking tools to investigate the binding of the predicted peptides to various MHC-I proteins. For example, Alam *et. al.*^45^ docked two predicted epitopes to HLA-A*53:01 with Autodock and reported good predicted binding affinities for the peptides. In none of these studies on ZIKV epitopes has MD been used to investigate the stability of the peptide-MHC-I complexes. On the other hand, there are several studies that have generally investigated the dynamics of peptide binding in the MHC-I binding groove by means of MD simulations (e.g. refs 39, 48 and 49). For example Fleischmann *et al*. studied the mechanism of how tapasin, a chaperone, takes part in controlling the quality of the binding peptides (high or low affinity binders)^50^. Their study suggests that a high-affinity peptide succeeds to close the binding groove tightly, while a low-affinity peptide (or the absence of a peptide) widens the groove. In our study, two of the predicted epitope peptides were closing the groove as they reduced the F pocket size (Supporting Information, Table S1): MAEVRSYCY (from ZIKV E protein) and IAMTDTTPY (from ZIKV NS5 protein). The rest of the peptides widened the groove to variable extent.

In case of linear epitopes, the leucine residues at positions 80 and 81 from the predicted ZIKV-NS3 antigenic site were also predicted as comprising a CTL epitope, thus significantly enhancing the possibility of this peptide to become a potential peptide vaccine candidate. Moreover, surface accessibility, surface flexibility as well as hydrophilicity of epitopes for E protein, NS3 and NS5 have been reported in the current study. The stability of the peptide-MHC I complexes has also been examined in the current study by MD simulations. Overall, the complexes get stabilized during the MD simulation after the favorable interactions have been formed and the possible steric clashes resulting from the rigid-body docking have been removed. Most of the electrostatic and hydrogen binding interactions are formed between the N- and C-terminal ends of the peptides, which is in agreement with the well-known pattern of epitope binding to MHC-I proteins. However, the PEP-FOLD3 predicted conformations of the peptides can be possible in solution but when the peptides bind to the binding groove, the experimental structures show a rather elongated conformation, C-terminus residing mainly in the more flexible F pocket and N-terminus in the less flexible pocket at the other end of the groove and some anchor residues in between interacting with the groove. In this case, flexible docking methods might work better when trying to dock the epitope peptides to the MHC-I protein binding groove in the described way.

Structural and genomic data, alongside the drastic development of vast genome sequence databases, aids in the design and discovery of novel vaccine candidates when coupled together with computational tools. Infection of ZIKV is a serious problem concerning morbidity and mortality worldwide. Unfortunately, the unavailability of vaccines against the ZIKV has affected many precious lives in various regions of the world. Researchers have beenstruggling to gather data associated to ZIKV to understand its biology, transmission and pathophysiology in order to eradicate the disease successfully. We inferred that the predicted epitopes possess therapeutic potential, with promising scope in the near future. Our immunoinformatics analysis will aid in the development of potential peptide vaccines using the predicted peptides.

## Materials and Methods

### ZIKV protein sequences

The primary amino acid sequences of ZIKV E, NS3, and NS5 proteins were retrieved from the ZIKV polyprotein sequence (GenBank ID: AHZ13508.1) that is deposited in the National Center for Biotechnology Information (NCBI) database (http://www.ncbi.nlm.nih.gov/protein/). The individual sequence lengths for the viral protein segments were 403 (E), 151 (NS3) and 641 (NS5).

### Prediction of Linear and Conformational B-Cell epitopes

Antigen B-cell epitope interacts with B-lymphocyte which causes the B-lymphocytes to differentiate into antibody-secreting plasma and memory cells^51^. B cell epitope has two important characteristics including hydrophilic nature and accessibility for a flexible region of an immunogen^52^. According to the Parker hydrophilicity prediction^33^, Emini prediction of surface accessibility^31^, Kolaskar and Tongaonkar antigenicity scale^30^, Karplus and Schulz Flexibility Prediction^32^ the analysis was performed computationally at IEDB (http://www.iedb.org/) analysis resource. ElliPro^38^ from IEDB analysis resource was employed for prediction of the discontinuous (conformational) epitopes for B-cell (http://tools.immuneepitope.org/toolsElliPro/). Three different algorithms are used in this resource including Protrusion Index (PI) of residues^53^, neighboring residues clustering depending upon PI and protein shape approximation^54^.

### Prediction of Potential Cytotoxic T-lymphocyte (CTL) epitopes

Using the NetCTL.1.2 server^36^ (http://www.cbs.dtu.dk/services/Net CTL), CTL epitopes were predicted. The activation of CTLs takes place on the surface of antigen-presenting MHC molecules. The prediction of the MHC class I binding, TAP transport efficiency and the proteasomal C-terminal cleavage was integrated with the help of NetCTL 1.2 server. As an input, the FASTA sequence of the organism was provided. Human leukocyte antigen (HLA) alleles and peptide lengths were both selected and submitted. As an output, T-cell epitopes were predicted. In order to predict proteasomal C-terminal cleavage and MHC class I binding, the artificial neural network was used while a weight matrix was used to predict the TAP transport efficiency.

### Homology modeling and structure validation

Homology modeling was performed for E protein, NS3 and NS5 proteins of ZIKV^37^. The Protein Data Bank (PDB) repository was employed to find suitable templates to generate the three-dimensional (3D) coordinate structures for all three protein sequences that were taken from UniProt (www.uniprot.org). PSI-BLAST^55^ was used to search for the homologous proteins from the PDB. ALIGN2D module was used to generate the initial alignment between the target and the template. Using a restrained-based approach in MODELLER v. 9.12, the 3D structures of all three proteins (E-protein, NS3, and NS5) were generated^56^. Moreover, spatial limitations were calculated with stereochemistry by CHARMM^57^. The stereochemical quality and correctness of the modelled structures was verified with WhatIF^58^, PROCHECK^59^ and Verify 3D^60^.

### Peptide-MHC protein complex and molecular docking studies

The predicted CTL epitope peptides of ZIKV E, NS3 and NS5 proteins that contained antigenic amino acid residues were selected for the docking analysis. 3D structures of these peptides were modeled with the PEP-FOLD3 server^61^, using 200 simulation runs to sample the conformations. After PEP-FOLD3 had clustered the different conformational models, they were sorted using the sOPEP energy^62^ value. Subsequently, the best ranked peptide models were docked to the selected class I MHC molecules including HLA-A (PDB ID: 2GIT), HLA-B (PDB ID: 2BST) and HLA-C (PDB ID: 4NT6), using the PatchDock rigid-body docking server^63^,^64^. PatchDock calculates potential complexes of two given molecules. All complexes that exhibit undesirable penetrations of the atoms of the receptor to the ligand are discarded and the remaining complexes are categorized according to the geometric shape complementarity score^63^,^64^. The docking results were then refined and scored with the FireDock server^65^,^66^. FireDock assists in resolving the flexibility and scoring problems produced by fast rigid-body docking programs^67^. The hydrogen bonding interactions of the docked structures were studied with the molecular visualization programs UCSF Chimera 1.11^68^ and PyMOL^69^ (Schrödinger, Inc).

### Molecular dynamics simulations

The stability of the docked complexes was further investigated by molecular dynamics (MD) simulations using the AMBER 12 simulation package^70^. After a stepwise minimization and equilibration protocol the solvated system (with TIP3P^71^ water) was submitted to a production simulation of 5 ns at 300 K and at 1 bar pressure (see Supporting Information for the detailed protocol). The ptraj module of AMBER was used for the trajectory analysis.

## Additional Information

**How to cite this article**: Usman Mirza, M. *et al*. Towards peptide vaccines against Zika virus: Immunoinformatics combined with molecular dynamics simulations to predict antigenic epitopes of Zika viral proteins. *Sci. Rep.* **6**, 37313; doi: 10.1038/srep37313 (2016).

**Publisher's note:** Springer Nature remains neutral with regard to jurisdictional claims in published maps and institutional affiliations.

## Supplementary Material

Supplementary Information

## Figures and Tables

**Table 1:** Predicted antigenic B-cell epitopes of ZIKV E protein*.

No.	Start position	End position	Peptide	Peptide length
1	20	34	WVDVVLEHGGCVTVM	15
2	38	49	KPTVDIELVTTT	12
3	56	64	VR**SYCY**EAS	9
4	71	77	DSRCPTQ	7
5	88	99	**TQYVCK**RTLVDR	12
6	110	123	KGSLVTCAKFACSK	14
7	138	147	RIMLSVHGSQ	10
8	185	191	SLGLDCE	7
9	198	204	**FSDLYYL**	7
10	210	216	**HWLV**HKE	7
11	219	226	HDIPLPWH	8
12	240	246	EALVEFK	7
13	252	260	RQTVVVLGS	9
14	262	273	EGAVHTALAGAL	12
15	285	294	SSGHLKCRLK	10
16	300	317	LKGVSYSL**CTAAFTFTKI**	18
17	323	333	H**GTVTVEVQY**A	11
18	338	346	PCKVPAQMA	9
19	352	358	LTPVGRL	7
20	360	367	TANPVITE	8
21	383	391	GDSYIVIGV	9

^*^21 antigenic sites were identified from the ZIKV E protein. Residues (in bold) were also predicted as CTL epitopes.

**Table 2:** Predicted antigenic B-cell epitopes of ZIKV NS3 protein*.

No.	Start position	End position	Peptide	Peptide length
1	13	33	LLGSTQVGVGVMQEGVFHTMW	21
2	56	66	KQDLVSYCGPW	11
3	76	86	**SEVQLLAV**PPG	11
4	92	98	IQTLPGI	7
5	106	114	**IGAVALDY**P	9
6	120	132	SPILDKCGRVIGL	13

^*^6 antigenic sites were identified from the ZIKV NS3 protein. Residues (in bold) were also predicted as CTL epitopes.

**Table 3:** Predicted antigenic B-cell epitopes of ZIKV’ NS5 protein*.

No.	Start position	End position	Peptide	Peptide length
1	13	21	RAVVSCAEA	9
2	67	91	ASSLINGVVRLLSKPWDVVTGVTGI	25
3	101	107	**Q**QRVFKE	7
4	140	147	RPRVCTKE	8
5	181	186	FWALVD	6
6	194	203	RGECQSCVYN	10
7	231	241	ARFLEFEALGF	11
8	262	270	LQRLGYVLE	9
9	312	322	R**ALALAIIKY**T	11
10	324	333	QNKVVKVLRP	10
11	353	361	GQVVTYALN	9
12	364	371	**TNLVVQLI**	8
13	413	421	GDDCVVKPI	9
14	426	431	AHALRF	6
15	457	470	VPFCSHHFNKLHLK	14
16	473	484	RSIVVPCRHQDE	12
17	488	494	RARVSPG	7
18	501	509	**ETACLAKSY**	9
19	512	520	**MWQLLY**FHR	9
20	528	539	NAICSSVPVDWV	12
21	560	566	**MLVV**WNR	7
22	597	604	WCGSLIGH	8

^*^22 antigenic sites were identified from the ZIKV NS5 protein. Residues (in bold) were also predicted as CTL epitopes.

**Table 4:** Predicted CTL epitopes from the ZIKV E protein.

Residue number	Peptide sequence	Predicted MHC binding affinity	Rescale binding affinity	C-terminal cleavage affinity	Transport affinity	Prediction score*	MHC ligand
53	MAE**VRSYCY**	0.4403	1.8696	0.9649	3.021	2.1654	Yes
85	QSD**TQYVCK**	0.2076	0.8813	0.5921	0.443	0.9922	Yes
129	SIQPENLEY	0.4389	1.8634	0.9329	3.175	2.1621	Yes
159	ETDENRAKV	0.1722	0.7311	0.9755	−0.11	0.872	Yes
195	GLD**FSDLYY**	0.6604	2.8041	0.7805	2.599	3.0511	Yes
198	**FSDLYYL**TM	0.5872	2.4933	0.9307	−0.065	2.6297	Yes
205	TMNNK**HWLV**	0.2026	0.8603	0.9214	0.369	1.0169	Yes
308	**CTAAFTFTK**	0.1876	0.7964	0.6855	0.467	0.9226	Yes
324	**GTVTVEVQY**	0.1984	0.8423	0.9769	2.779	1.1278	Yes
368	STENSKMML	0.2251	0.9558	0.9741	0.675	1.1357	Yes

^*^Prediction score threshold was set at >0.75000. Bold indicates amino acids that were also predicted as antigenic sites.

**Table 5:** Predicted CTL epitopes from the ZIKV NS3 protein.

Residue number	Peptide sequence	Predicted MHC binding affinity	Rescale binding affinity	C-terminal cleavage affinity	Transport affinity	Prediction score*	MHC ligand
1	TTDGVYRVM	0.1514	0.643	0.9115	0.084	0.7839	Yes
47	RLDPYWGDV	0.1625	0.6897	0.9687	0.372	0.8536	Yes
75	H**SEVQLLAV**	0.277	1.176	0.2538	0.09	1.2186	Yes
105	D**IGAVALDY**	0.2072	0.8797	0.9491	2.544	1.1492	Yes
136	GVVIKNGSY	0.1489	0.6321	0.9462	3.103	0.9292	Yes

^*^Prediction score threshold was set at >0.75000. Bold indicates amino acids that were also predicted as antigenic sites.

**Table 6:** Predicted CTL epitopes fromthe ZIKV NS5 protein.

Residue number	Peptide sequence	Predicted MHC binding affinity	Rescale binding affinity	C-terminal cleavage affinity	Transport affinity	Prediction score*	MHC ligand
51	RTWAYHGSY	0.3114	1.3221	0.9789	3.267	1.6323	Yes
67	ASSLINGVV	0.1418	0.6019	0.8948	0.549	0.7636	Yes
91	**I**AMTDTTPY	0.1612	0.6844	0.8903	3.035	0.9697	Yes
93	MTDTTPYG**Q**	0.2728	1.1583	0.1576	−0.102	1.1769	Yes
124	MSMVSSWLW	0.2011	0.8539	0.8448	1.084	1.0349	Yes
233	FLEFEALGF	0.1492	0.6333	0.3368	2.447	0.8062	Yes
313	**ALALAIIKY**	0.216	0.9172	0.9296	3.237	1.2185	Yes
315	**ALAIIKYTY**	0.2376	1.0088	0.9516	3.153	1.3092	Yes
363	**FTNLVVQLI**	0.1698	0.7208	0.4951	0.334	0.8118	Yes
501	**ETACLAKSY**	0.3732	1.5844	0.3155	2.848	1.7742	Yes
509	**Y**AQ**MWQLLY**	0.6788	2.8819	0.8387	2.979	3.1567	Yes
555	MTTED**MLVV**	0.1969	0.8361	0.2914	0.248	0.8922	Yes
581	VTKWTDIPY	0.2405	1.0211	0.8992	2.818	1.2969	Yes
584	WTDIPYLGK	0.3037	1.2893	0.9376	0.341	1.447	Yes
631	YMDYLSTQV	0.288	1.223	0.9517	0.341	1.3828	Yes

^*^Prediction score threshold was set at >0.75000. Bold indicates amino acids that were also predicted as antigenic sites.

**Table 7:** Homology modeling parameters.

ZIKV target proteins	Template PDB ID (chain)	Description	Query Cover	E-Value	Positives	Maximum Identity
**E**	3G7T (A)	Crystal structure of dengue virus type 1 envelope protein	99%	1.00E-167	72%	57%
	1UZG (A)	Crystal structure of dengue virus type 3 virus envelope protein	99%	2.00E-164	72%	57%
	4GSX (A)	Crystal structure of Japanese Encephalitis virus envelope protein	100%	3.00E-164	72%	52%
	2I69 (A)	Crystal structure of West Nile virus envelope glycoprotein	100%	3.00E-167	72%	52%
**NS3**	2YOL (A)	West Nile virus NS2B-NS3 protease in complex with 3,4-dichloro-phenylacetyl-Lys-Lys-GCMA	100%	1.00E-73	81%	70%
	2WV9 (A)	Crystal structure of the NS3 protease-helicase from Murray Valley encephalitis virus	100%	8.00E-65	80%	66%
	4R8T (B)	Structure of JEV protease	90%	1.00E-60	78%	63%
**NS5**	2HFZ (A)	Crystal structure of RNA dependent RNA polymerase domain from West Nile virus	95%	0.00E + 00	84%	72%
	4K6M (A)	Crystal structure of the full-length Japanese encephalitis virus NS5	100%	0.00E + 00	82%	70%
	4V0Q (A)	Dengue virus full length NS5 complexed with SAH	99%	0.00E + 00	80%	68%

**Table 8:** Conformational epitopes of ZIKV-E protein as predicted by ElliPro.

No	Residues	No. of residues	Score
1	I65, S66, D67, M68, A69, S70, D71, S72, R73, C74, P75, T76, Q77, G78, E79, A80, Y81, L82, D83, K84, Q85, S86, D87, T88, Q89, Y90, C92, K93, R94, T95, L96, V97, D98, R99, G100, W101, G102, N103, G104, C105, G106, L107, F108, G109, K110, G111, S112, L113, V114, T115, C116, A117, K118, D230, T231, T233, P234, H235, E244, F245, K246, D247, A248, H249, A250, K251, R252, Q253, T254, V255, V256	71	0.744
2	D10, F11, V12, E13, G14, M15, S16, G17, G18, T19, W20, M34, A35, Q36, D37, K38, P39, T40, V143, H144, G145, S146, Q147, H148, S149, G150, M151, I152, V153, N154, D155, T156, G157, H158, E159, T160, D161, E162, N163, R164, A165, T170, P171, N172, S173, P174, R175, A176, E177, A178, T179, L180, G181, G182, F183, G184, S185, L186, G187, D189, R292, K294, M295, D296, K297, L298, R299, L300, K301	69	0.728
3	I1, S306, L307, C308, T309, A310, A311, F312, T313, F314, T315, K316, I317, P318, V330, Q331, Y332, A333, G334, T335, D336, G337, P338, C339, K340, V341, P342, A343, Q344, M345, A346, V347, D348, M349, Q350, T351, L352, T353, P354, V355, G356, R357, L358, V364, I365, T366, E367, S368, T369, E370, N371, S372, M374, L378, D379, P380, P381, F382, G383, D384, S385, Y386, I387, V388, I389, G390, V391, G392, E393, K394, K395, I396, T397, H398, H399, W400, H401, R402, S403	79	0.697

**Table 9:** Conformational epitopes of ZIKV-NS3 protein as predicted by ElliPro.

No	Residues	No. of residues	Score
1	T10, R11, R12, L13, L14, G15, S16, T17	8	0.871
2	V137, V138, I139, K140, N141, G142, S143	7	0.855
3	W72, D73, G74, H75, S76, E77, Q93, L95, P96, G97, I98, F99, K100, T101, K102, D103, G104, D105	18	0.727
4	R88, A89, R90, N91	4	0.707
5	T1, T2, D3, E26, G27, G38, S39, A40, R42, S43, G44, E45, G46, R47, L48, D49, P50, Y51, C63, G64, P65, W66, K67, L68	24	0.662
6	P84, P85, G86, E87	4	0.629

**Table 10:** Conformational epitopes of ZIKV-NS5 protein as predicted by ElliPro.

No	Residues	No. of residues	Score
1	N72, G73, V74, R76, L77, L78, S79, K80, P81, D83, V84, V85, T86, K466, L467, H468, L469, K470, D471, G472, R473, S474, R490, R500, F518, R521, L525, N528, A529, C531, S532, S533, V534, P535, V536, D537, W538, T556, T557, E558, D559, L561, V562, N565, R566, I569, E570, E571, N572, D573, H574, M575, E576, D577, K578, T579, P580, V581, T582, K583, W584, T585, D586, I587, P588, Y589, L596, G599, S600, L601, I602, G603, H604, P606, R607, T608, T609, W610, A611, E612, N613, I614, K615, N616, T617, V618, N619, M620, V621, R622, I624, I625, G626, D627, E628, E629, K630, Y631, M632, D633, Y634, L635, S636, T637, Q638, V639, R640, Y641	108	0.736
2	E3, E4, D5, V6, N7, L8, G9, S10, G11, T12, R13, A14, V15, V16, S17, C18, A19, E20, A21, P22, N23, M24, I26, I27, G28, N29, I31, E32, R33, I34, R35, S36, E37, H38, A39, E40, T41, W42, F43, F44, D45, E46, N47, H48, P49, Y50, R51, Y55, H56, G57, S58, Y59, E60, A61, P62, T63, Q64, T97, Y99, D110, R140, P141, R142, V143, C144, T145, K146, E147, I150, R154, E165, E166, K167, E168, W169, T171, V173, E174, A175, V176, N177, D178, P179, R180, F181, W182, A183, L184, V185, D186, K187, E188, R189, E190, H191, H192, L193, R194, G195, E196, C197, Q198, S199, C200, E237, T304, N305, Q306, M307, E308, K309, G310, H311, R312, A313, L314, L316, Y321, N325, R332, P333, A334, E335, K336, G337, K338, T339, V340	128	0.715
3	P116, Q117, E118, G119, T120, R121, Q122, V123, S125, M126, S129, W130, K133, E134, G136, K137, H138, K139, W247, S273, R274, I275, P276, G277, G278, R279, M280, Y281, N373, M374, E375, A376, E377, E378, V379, L380, E381, M382, Q383, D384, L385, W386, L387, L388, R389, R390, S391, E392, K393, V394, T395, N396, W397, L398, Q399, S400, N401, G402, W403, D404, R405, R408, K419, P420, I421, D422, D423, R424, A426, H427, A428, L429, R430, F431, D434, M435, T442, Q443, E444, W445, K446, P447, S448, T449, G450, W451, D452, N453	88	0.703
4	L590, G591, K592, R593, E594, W597	6	0.621
5	G494, A495, G496, W497, S498, I499	6	0.52

**Table 11:**
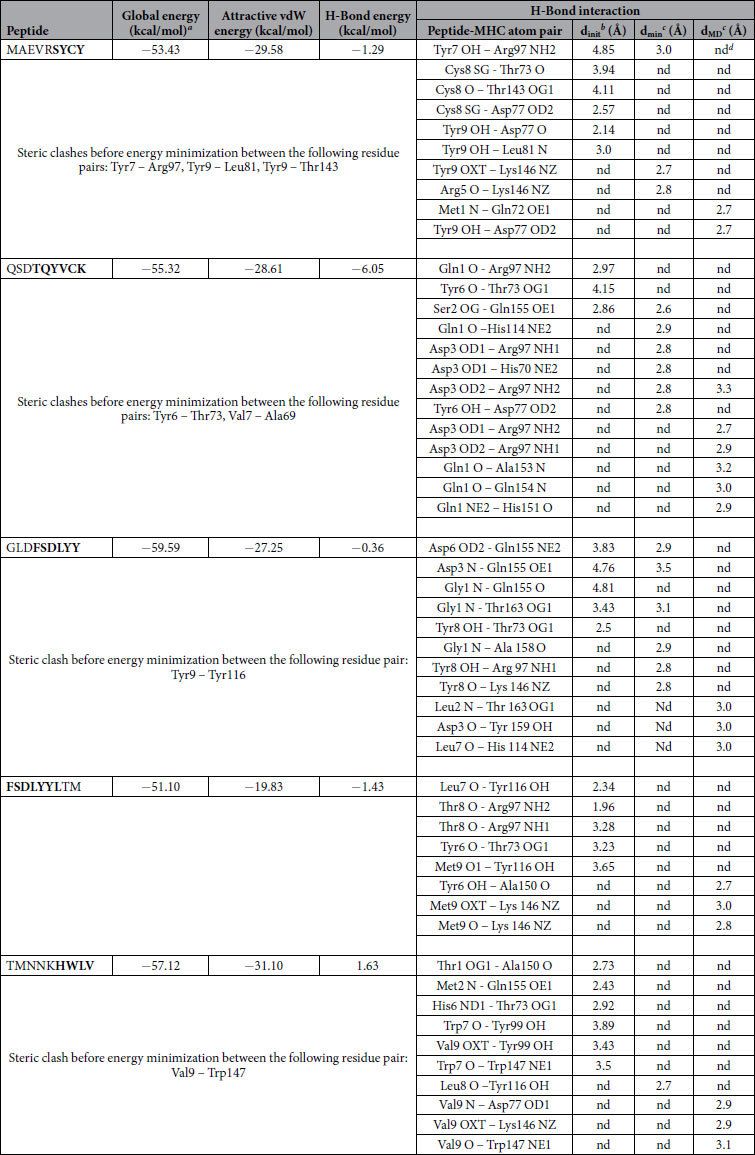
Zika E peptides–HLA-A*02:01 interaction.

^a^FireDock energy for the best ranked complex

^b^initial distance between the H-bond donor and the acceptor; measured with the FindHBond tool in Chimera (H-Bond constraints were relaxed by 1 Å and 20.0 degrees)

^c^distance between the H-bond donor and the acceptor after the energy minimization (min) and after the molecular dynamics simulation (MD); measured in PyMOL

^d^nd = no detected H-bond.

**Table 12:**
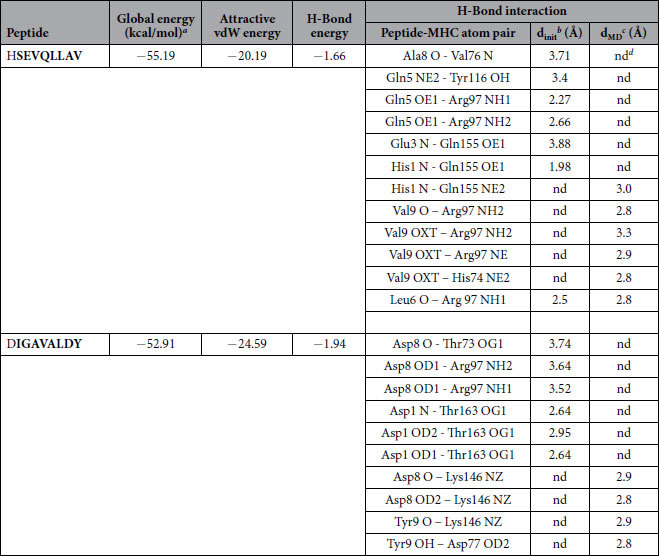
Zika NS3 peptides–HLA-B*27:05 interaction.

^a^FireDock energy for the best ranked complex.

^b^initial distance between the H-bond donor and the acceptor; measured with the FindHBond tool in Chimera (H-bond constraints were relaxed by 1 Å and 20.0 degrees).

^c^distance between the H-bond donor and the acceptor after the molecular dynamics simulation (d_MD_); measured in PyMOL.

^d^nd = no detected H-bond.

**Table 13:**
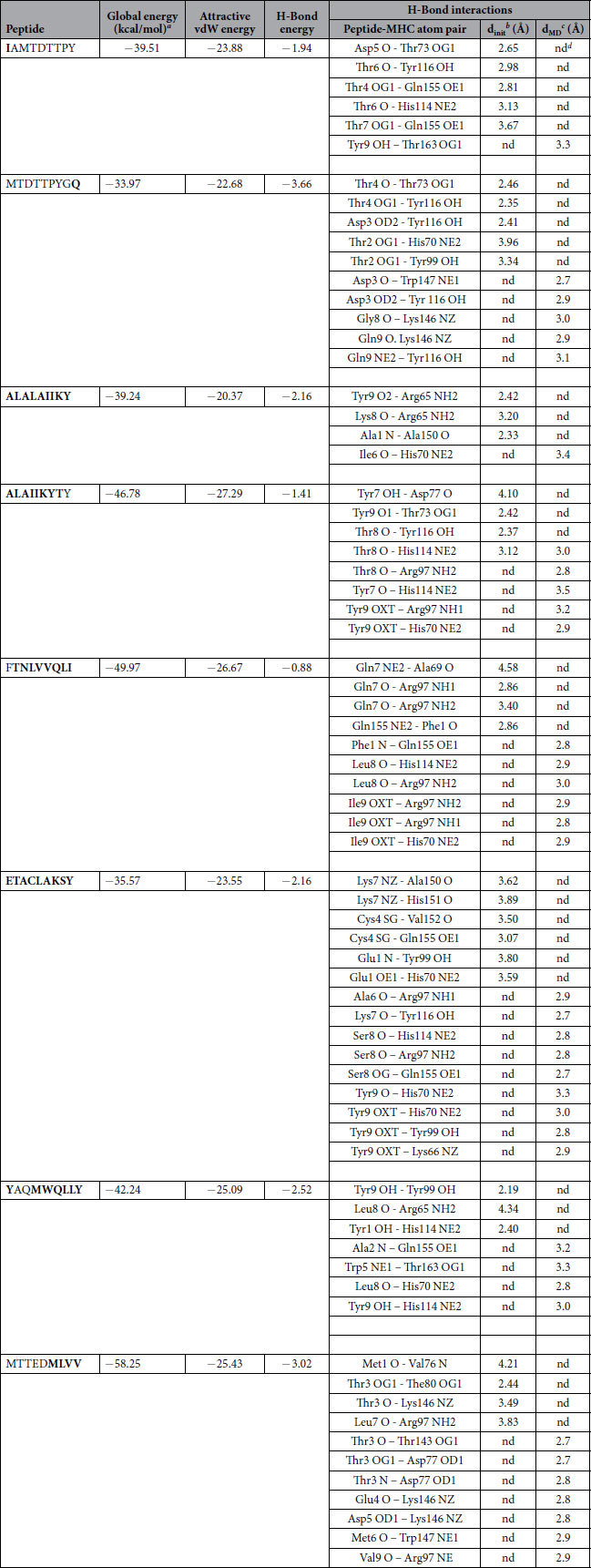
Zika NS5 peptides – HLA-C*08:01 interaction.

^a^FireDock energy for the best ranked complex.

^b^initial distance between the H-bond donor and the acceptor; measured with the FindHBond tool in Chimera (H-bond constraints were relaxed by 0.4 Å and 20.0 degrees).

^c^distance between the H-bond donor and the acceptor after the molecular dynamics simulation (d_MD_); measured in PyMOL.

^d^nd = no detected H-bond.
