# Erratum: Towards peptide vaccines against Zika virus: Immunoinformatics combined with molecular dynamics simulations to predict antigenic epitopes of Zika viral proteins

**DOI:** 10.1038/srep44633

**Published:** 2017-04-11

**Authors:** Muhammad Usman Mirza, Shazia Rafique, Amjad Ali, Mobeen Munir, Nazia Ikram, Abdul Manan, Outi M. H. Salo-Ahen, Muhammad Idrees

Scientific Reports 6: Article number: 3731310.1038/srep37313; published online: 12 09 2016; updated: 04 11 2017

In this Article, an affiliation was omitted for Muhammad Usman Mirza. The correct affiliations are listed below:

Center for Research in Molecular Medicine (CRiMM), University of Lahore, Pakistan.

Department of Pharmaceutical and Pharmacological Sciences, Rega Institute for Medical Research, Medicinal Chemistry, University of Leuven, Leuven, Belgium.

The original version of this Article also incorrectly listed Muhammad Usman Mirza’s surname as Usman Mirza.

In addition, the ‘How to cite’ section now reads:

Mirza, M. U. *et al*. Towards peptide vaccines against Zika virus: Immunoinformatics combined with molecular dynamics simulations to predict antigenic epitopes of Zika viral proteins. *Sci. Rep*. **6**, 37313; doi: 10.1038/srep37313 (2016).

These errors have now been corrected in the HTML and PDF versions of the Article.

