# Supplementary material for: Towards peptide vaccines against Zika virus: Immunoinformatics combined with molecular dynamics simulations to predict antigenic epitopes of Zika viral proteins
Source: Sci Rep. 2016 Dec 9;6:37313. doi: 10.1038/srep37313 (PMC5146661; doi:10.1038/srep37313)
Supplement: Supplementary Information [file srep37313-s1.pdf]

Supporting Information

Towards peptide vaccines against Zika virus: Immunoinformatics combined with molecular dynamics simulations to predict antigenic epitopes of Zika viral proteins

Muhammad Usman Mirza (M.Phil)[1,2], Shazia Rafique (Ph.D)[3], Amjad Ali (Ph.D)[3], Mobeen Munir (PhD)[4], Nazia Ikram (M.Phil)[5], Abdul Manan (M.Phil)[5], Outi M. H. Salo-Ahen[6,7] Muhammad Idrees (Ph.D)[3, 8]*

[1]Center for Research in Molecular Medicine (CRiMM), University of Lahore, Pakistan
[2]Department of Pharmaceutical and Pharmacological Sciences, Rega Institute for Medical Research, Medicinal Chemistry, University of Leuven, Leuven, Belgium.

[3]Centre for Applied Molecular Biology, University of the Punjab, Lahore, Pakistan

[4]Division of Science and Technology, University of Education Lahore, Pakistan

[5]Institute of Molecular Biology and Biotechnology (IMBB), University of Lahore, Pakistan

[6]Structural Bioinformatics Laboratory, Faculty of Science and Engineering, Biochemistry, Åbo Akademi University, Turku, Finland

[7]Pharmaceutical Sciences Laboratory, Faculty of Science and Engineering, Pharmacy, Åbo Akademi University, Turku, Finland.

[8] Vice Chancellor Hazara University, Mansehra, Pakistan

*Corresponding author
Centre for Applied Molecular Biology, University of the Punjab, 87-West Canal Bank Road, Thokar Niazbaig, Lahore-53700, Pakistan; Tel: +92-42-5293141; Fax: +92-42-5293149; Email: MI: idrees.khan@pu.edu.pk

Email addresses: MUM: muhammad.usman@imbb.uol.edu.pk; SR: shazia.camb@pu.edu.pk; AA: amjad.camb@pu.edu.pk; NI: naxiaikram@gmail.com; MM: mmunir@ue.edu.pk; AM: abdul.manan@imbb.uol.edu.pk; MI: idrees.khan@pu.edu.pk; OMHS-A: outi.salo-ahen@abo.fi

Molecular dynamics simulation protocol

The stability of the docked complexes was studied with molecular dynamics (MD) simulations. Energy minimization and equilibration of the simulation system and standard production simulations were performed with the AMBER package (version 12)1 using the AMBER ff03 force field2. The tleap module of AMBER was used to prepare the simulations system. All simulations were run in an octahedral box extending 10.0 Å around the solute and filled with explicit TIP3P water molecules3 and neutralizing Na+-ions. Periodic boundary conditions, particle-mesh Ewald electrostatics4 and a cut-off of 9 Å for non-bonded interactions were applied. A time step of 1 fs (only for Langevin dynamics during equilibration) or 2 fs was used together with the SHAKE algorithm5 to constrain the bonds to hydrogen atoms. The 5-ns production simulations were performed at a constant temperature (300 K) and pressure (1 bar). The coupling constants for temperature and pressure6 were 5.0 and 2.0 ps, respectively. Energy minimization was performed with the steepest descent (first 10 iterations) and conjugate gradient methods (subsequent 190 iterations), gradually reducing the restraint force constant on the protein atoms from 10 to 0 kcal/molÅ2. The stepwise system equilibration was performed as follows: (i) 10 ps heating of the system from 10 K to 300 K with a Langevin thermostat (collision frequency γ = 1.0 ps-1), keeping the volume constant and restraint force constant as 5 kcal/molÅ2 on the protein atom positions); (ii) same as the first step but for 20 ps and without any positional restraints; (iii) 20 ps MD at 300 K using a Langevin thermostat (γ = 0.5 ps-1) and constant volume, without positional restraints; (iv) 50 ps MD at 300 K using a Langevin thermostat (γ = 0.5 ps-1) and constant pressure of 1.0 bar (coupling constant for pressure = 1.0 ps), no restraints on the protein; (v) 400 ps MD at 300 K and at constant pressure of 1 bar (coupling constant for temperature = 5.0 ps and for pressure = 2.0 ps), no positional restraints. The MD simulation trajectories were analyzed

with the ptraj module of AMBER. The final frame structures were minimized with AMBER in the same way as the last step of the initial minimization.

A

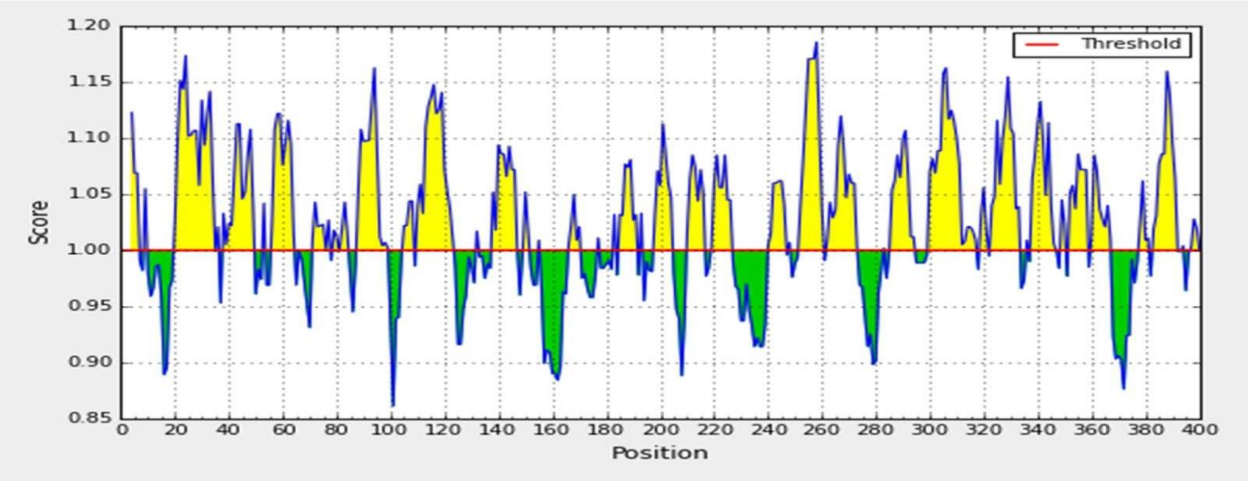

B

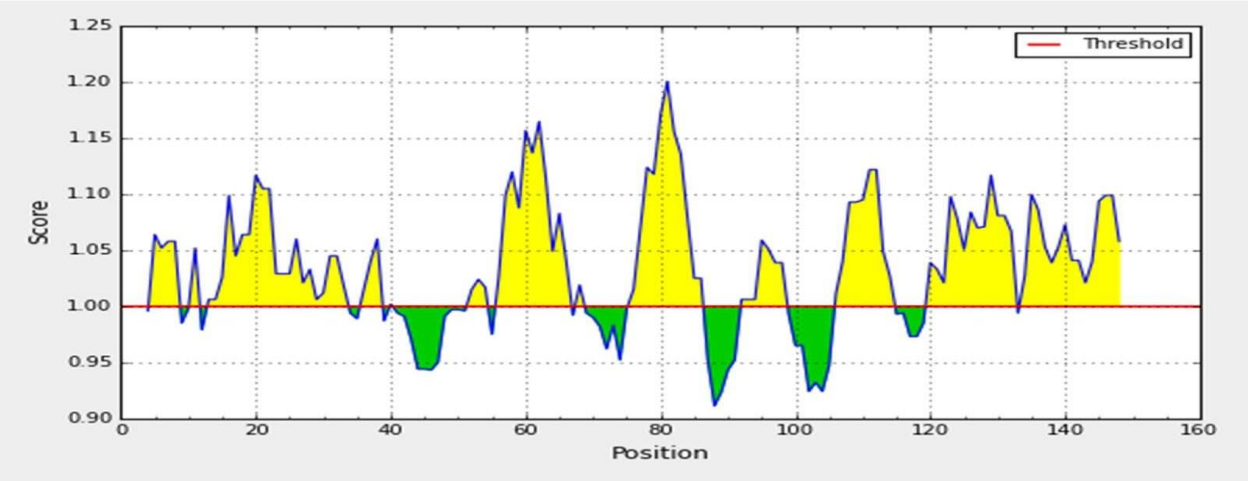

C

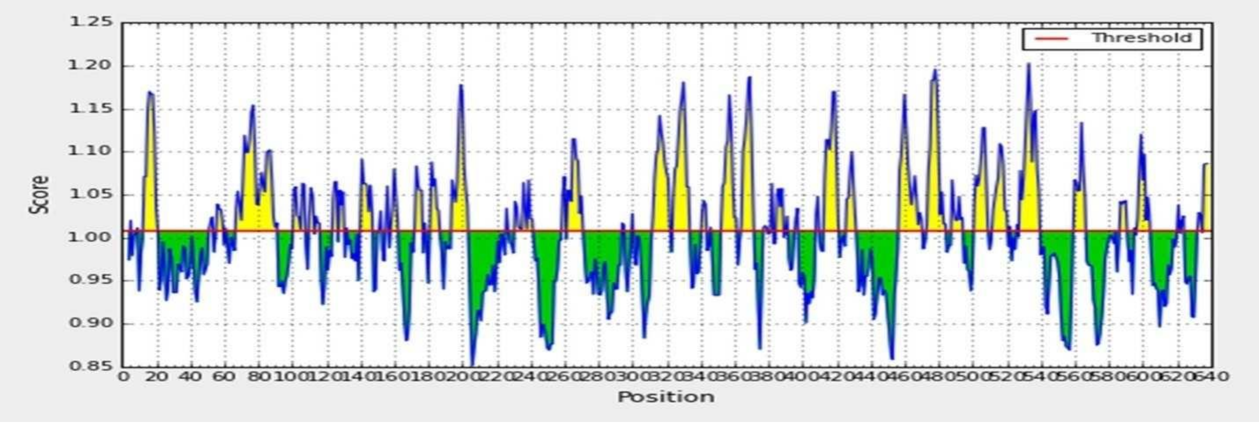

Fig. S1. Graphical representation of predicted antigenic propensity of E Protein (A), NS3 (B) and NS5 (C)

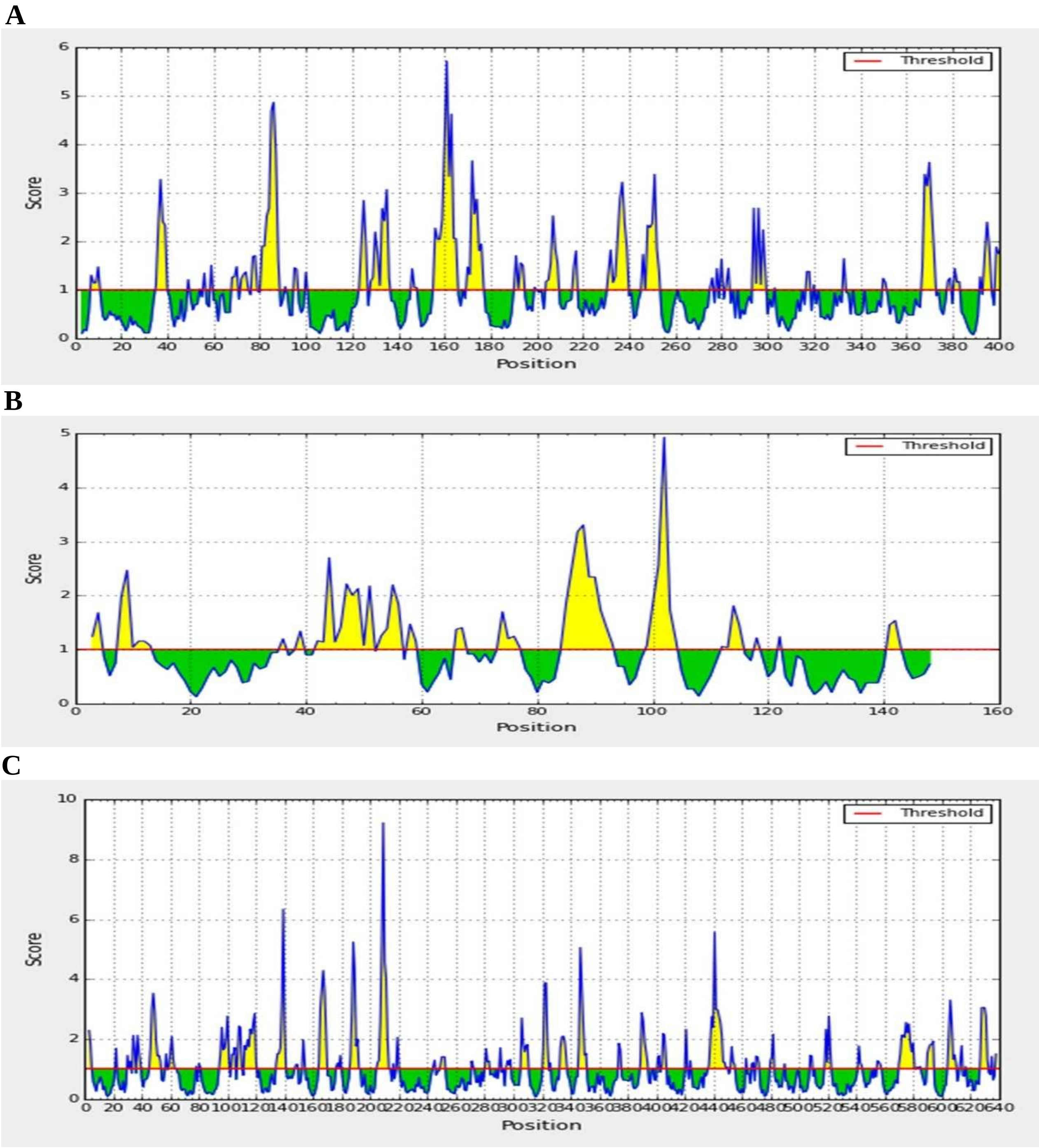

Fig. S2. Graphical representation of predicted surface probability of E Protein (A), NS3 (B) and NS5 (C)

A

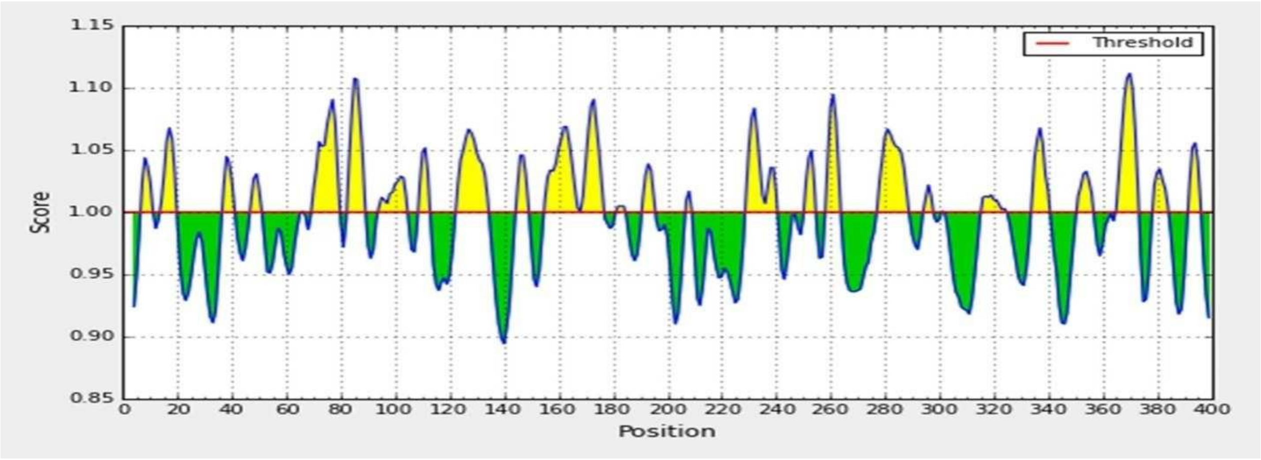

B

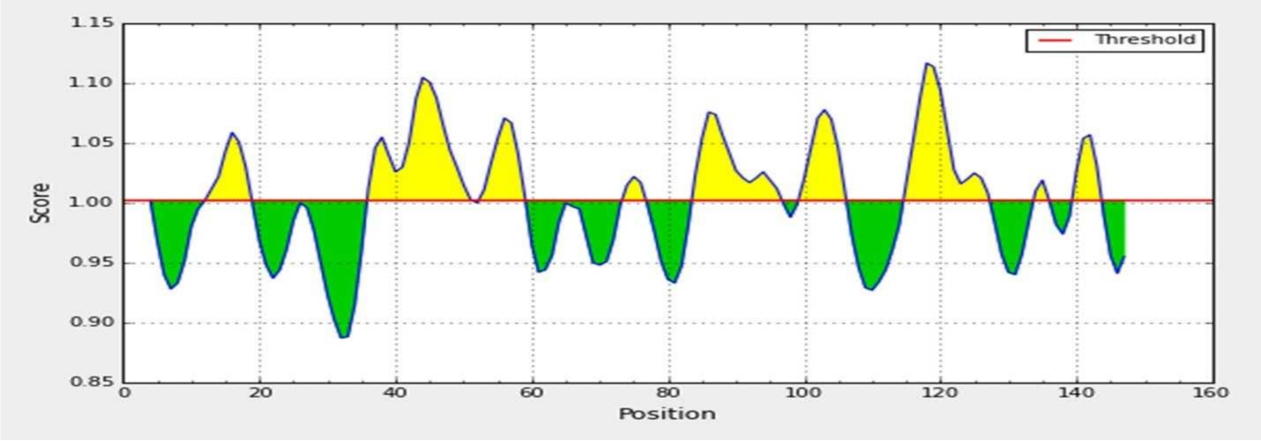

C

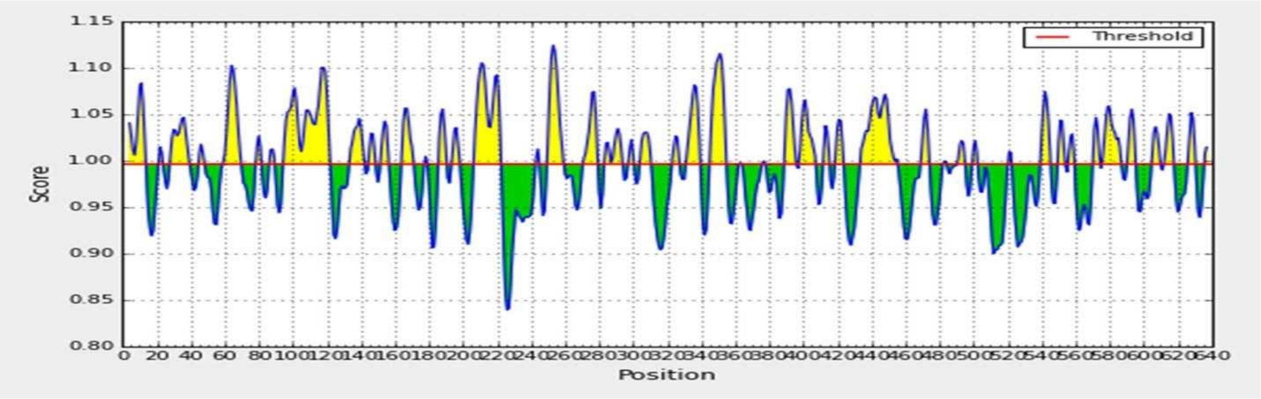

Fig. S3. Graphical representation of predicted surface flexibility of E Protein (A), NS3 (B) and NS5 (C)

A

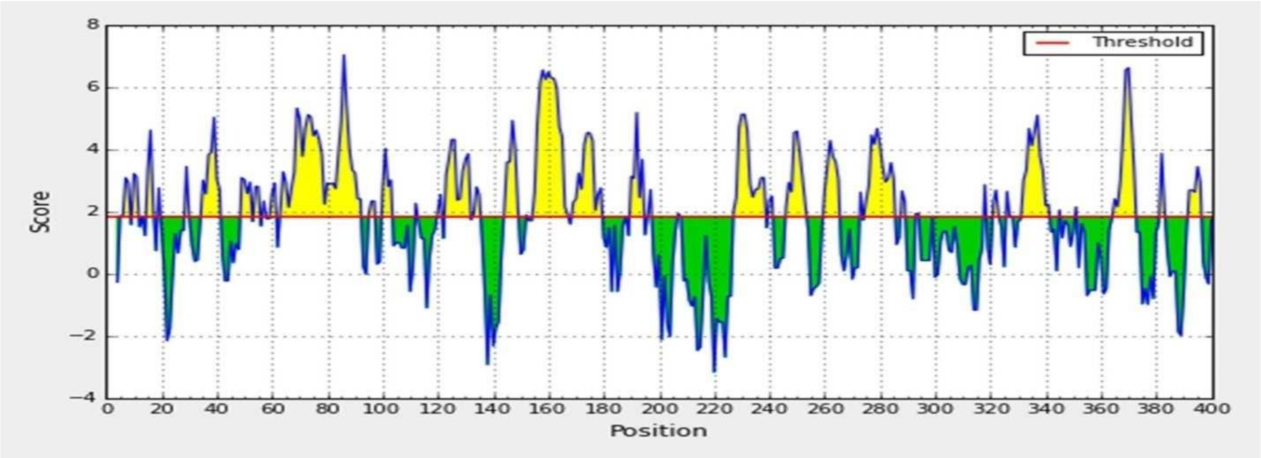

B

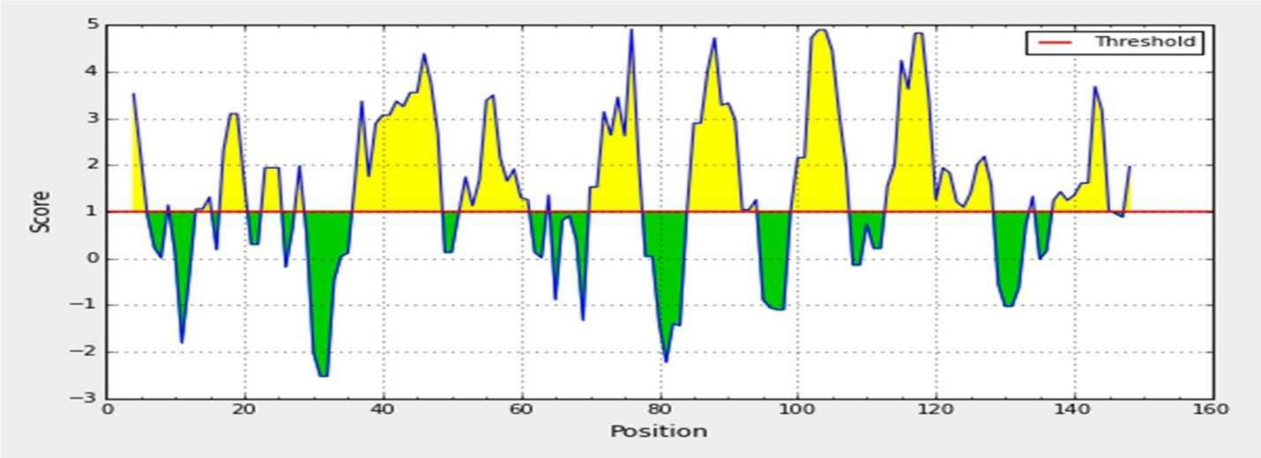

C

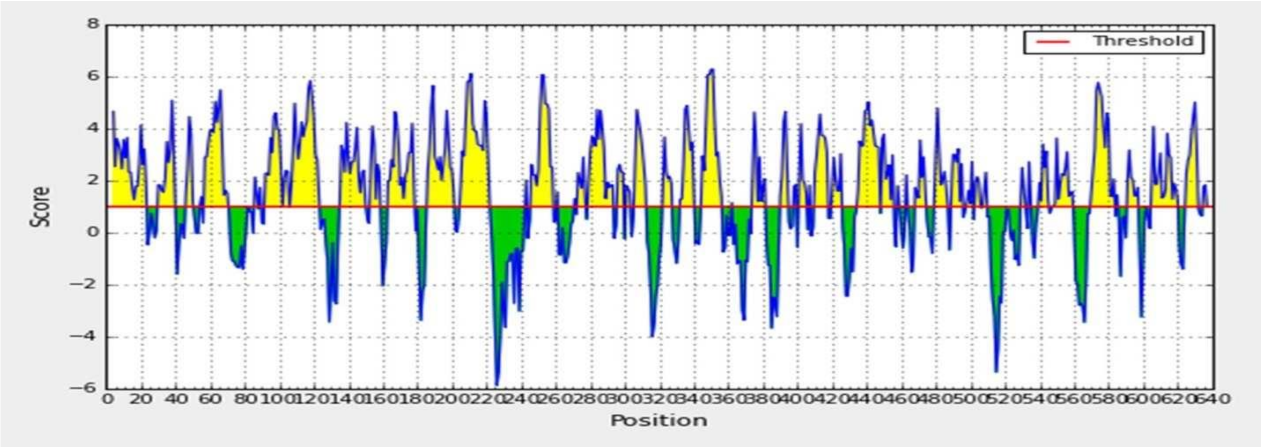

Fig. S4. Graphical representation of predicted surface hydrophilicity of E Protein (A), NS3 (B) and NS5 (C)

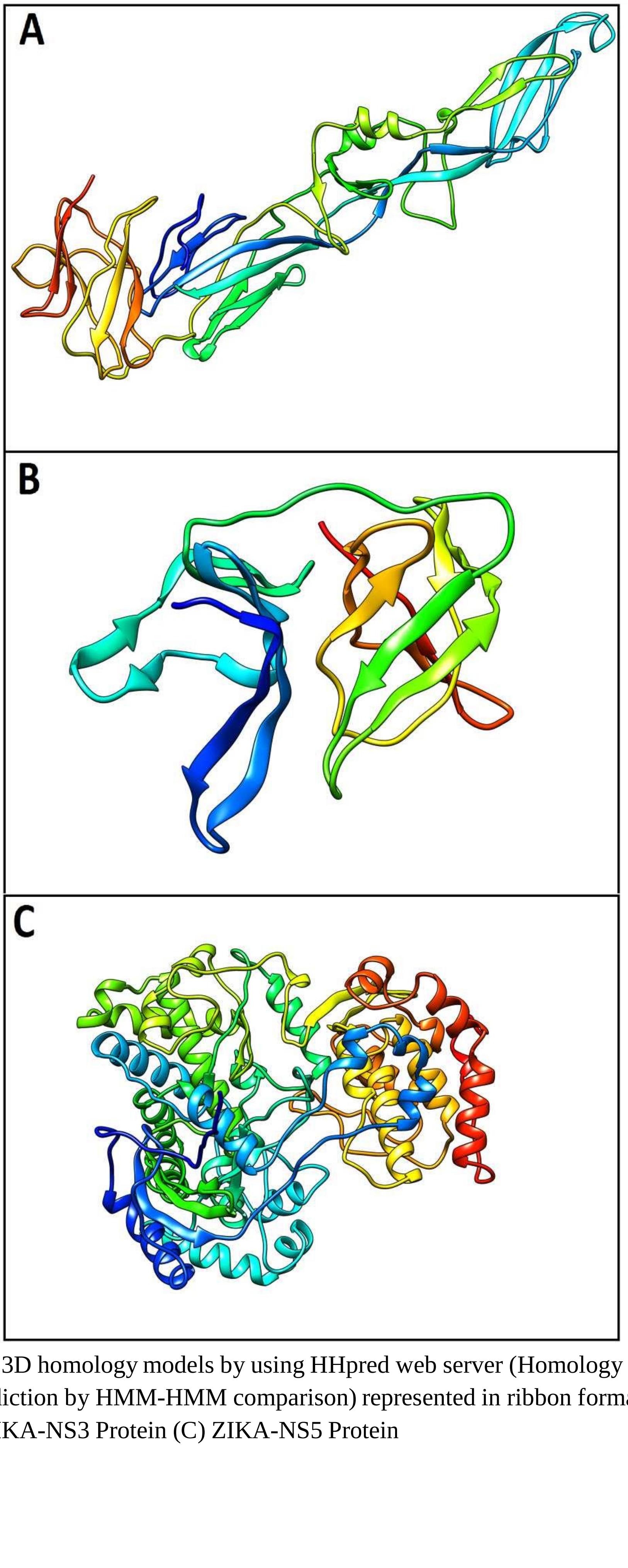

Fig. S5. Protein 3D homology models by using HHpred web server (Homology detection & structure prediction by HMM-HMM comparison) represented in ribbon format. (A) ZIKA-E Protein (B) ZIKA-NS3 Protein (C) ZIKA-NS5 Protein

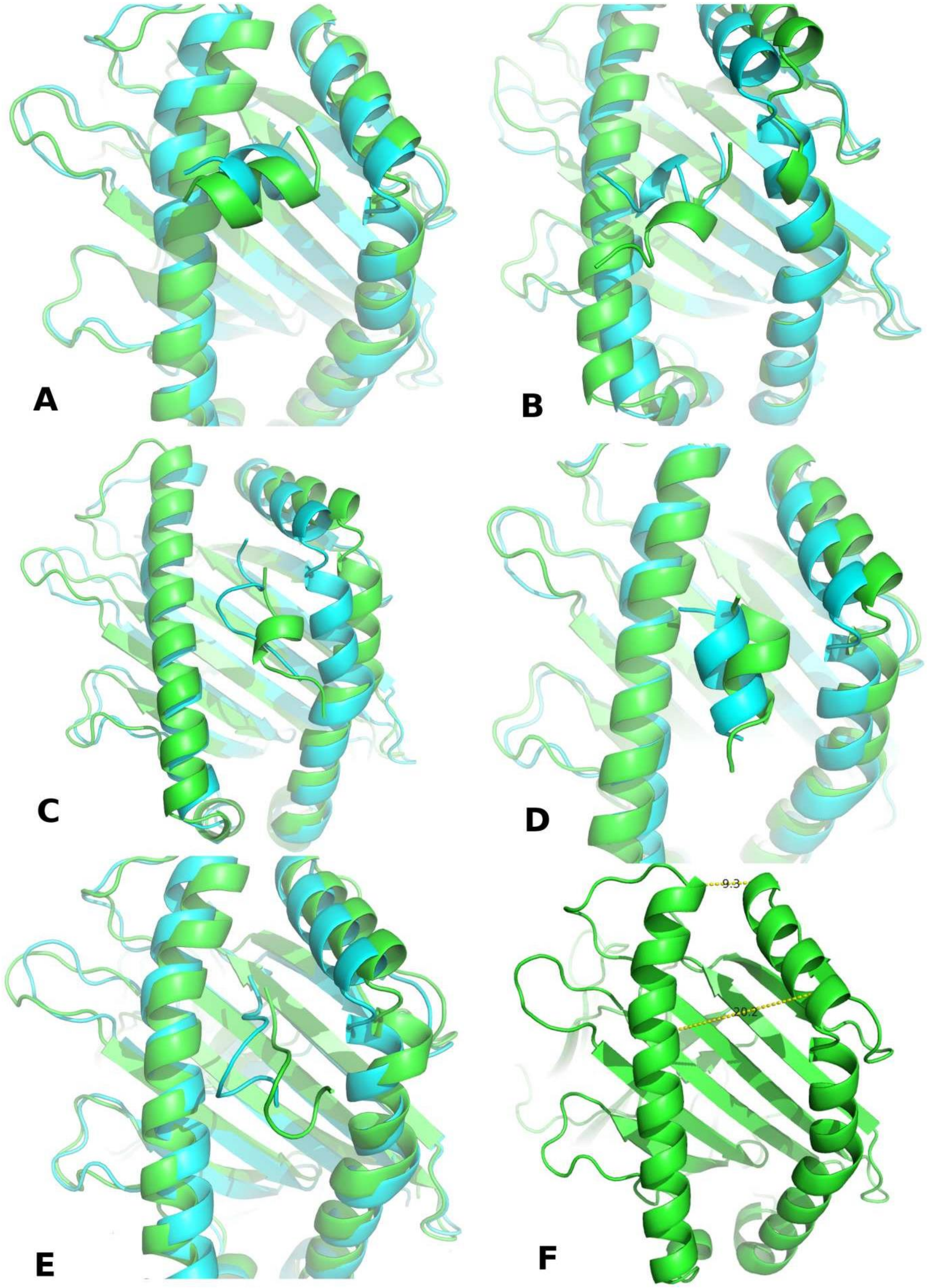

Fig. S6. ZIKV E peptide-MHC-I protein complexes (cartoon representation); the complex after energy minimization (in cyan) is superimposed with the complex after 5-ns MD simulation (in green): (A) MAEVRSYCY; (B) QSDTQYVCK; (C) GLDFSDLYY; (D) FSDLYYLTM; (E) TMNNKHWLV. (F) MHC-I (green cartoon, model before energy minimization) binding groove (F pocket) dimensions; distance 1 (shorter) and distance 2 (longer) are shown as yellow dashed lines.

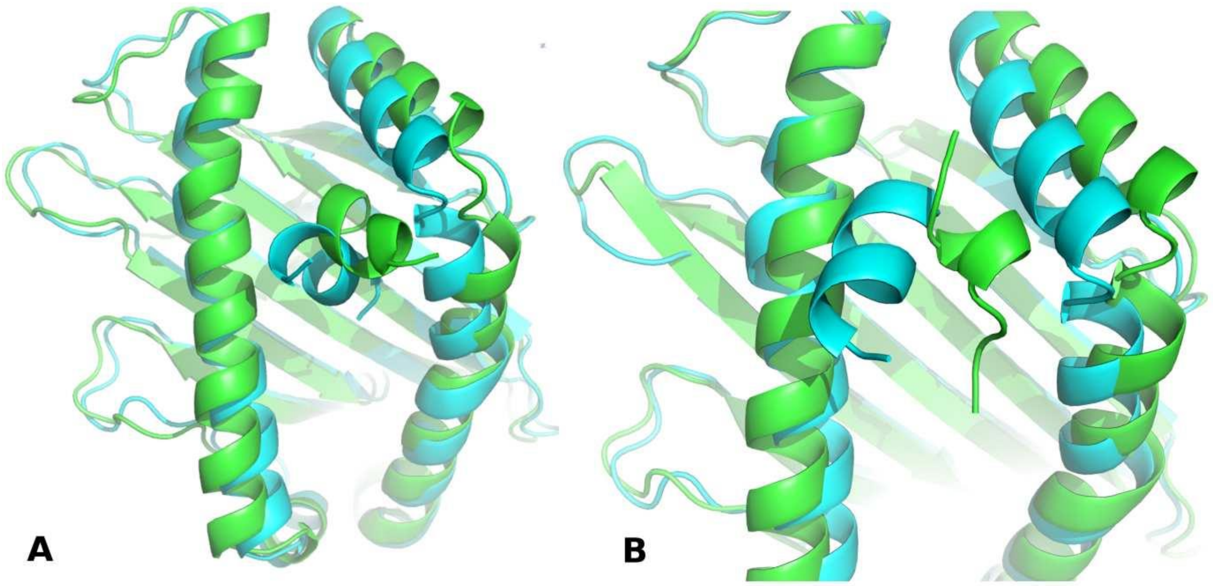

Fig. S7. ZIKV NS3 peptide-MHC-I protein complexes (cartoon representation); the complex after energy minimization (in cyan) is superimposed with the complex after 5-ns MD simulation (in green): (A) HSEVQLLAV; (B) DIGAVALDY.

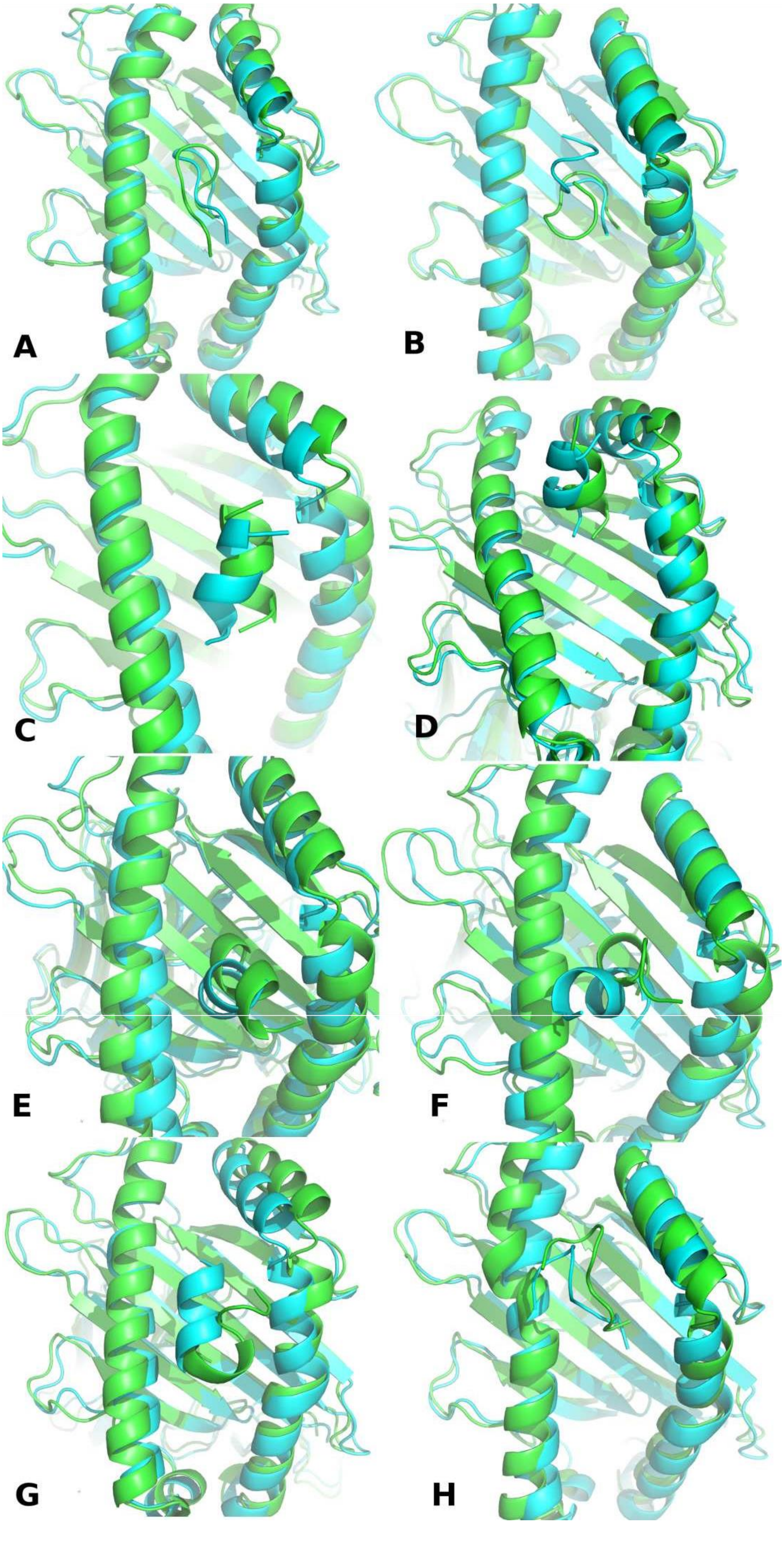

Fig. S8. ZIKV NS5 peptide-MHC-I protein complexes (cartoon representation); the complex after energy minimization (in cyan) is superimposed with the complex after 5-ns MD simulation (in green): (A) IAMTDTTPY (B) MTDTTPYGQ (C) ALALAIIKY (D) ALAIIKYTY (E) FTNLVVQLI (F) ETACLAKSY (G) YAQMWQLLY (H) MTTEDMLVV.

Table S1. Dynamics of the ZIKV peptide-MHC-I complexes

Peptide	Conformational change of the peptide (RMSD in Ångströms)[a]	Change in the MHC-I binding groove (F pocket) size	
		**d1/d2[b] (Å) (initial)**	**d1/d2 (Å) (after MD)**
MAEVRSYCY	0.597	9.1/21.4	**7.6/20.4**[c]
QSDTQYVCK	2.118	8.8/20.6	10.1/21.9
GLDFSDLYY	1.189	8.9/20.9	11.4/22.0
FSDLYYLTM	1.080	8.9/21.1	10.5/21.6
TMNNKHWLV	1.146	8.9/20.9	8.3/21.3
HSEVQLLAV	1.054 (8 atoms)	8.9/20.6	10.4/21.2
DIGAVALDY	3.105	9.0/21.3	10.4/23.5
IAMTDTTPY	1.043	8.8/20.5	**8.8/20.0**[c]
MTDTTPYGQ	1.760	8.9/20.6	10.7/21.4
ALALAIIKY	0.470	8.9/20.7	7.5/22.0
ALAIIKYTY	0.485 (8 atoms)	8.8/20.7	11.4/22.6
FTNLVVQLI	0.804	8.9/20.4	10.1/21.1
ETACLAKSY	1.372	9.0/20.6	11.4/21.1
YAQMWQLLY	1.481	8.9/20.5	11.3/23.0
MTTEDMLVV	1.690	8.8/20.8	11.1/23.4

[a] RMSD of the Cα atoms between the initial docked peptide conformation and the conformation in the final MD frame; [b]d1=distance between the Cα atoms of Tyr85 in α1 helix and Met138 in α2 helix; d2=distance between the Cα atoms of His74 in α1 helix and Ala149 in α2 helix; [c]F pocket size has been reduced during the MD simulations

References

1 Case, D. A. et al. The Amber biomolecular simulation programs. J. Comput. Chem. 26, 1668-1688, doi:10.1002/jcc.20290 (2005).

2 Duan, Y. et al. A point charge force field for molecular mechanics simulations of proteins based on condensed phase quantum mechanical calculations. J. Comput. Chem. 24, 1999-2012 (2003).

3 Jorgensen, W. L., Chandrasekhar, J., Madura, J. D., Impey, R. W. & Klein, M. L. Comparison of simple potential functions for simulating liquid water. J. Chem. Phys. 79, 926-935 (1983).

4 Essmann, U. et al. A smooth particle mesh Ewald method. J. Chem. Phys. 103, 8577-8593 (1995).

5 Ryckaert, J.-P., Ciccotti, G. & Berendsen, H. J. Numerical integration of the cartesian equations of motion of a system with constraints: molecular dynamics of n-alkanes. J. Comput. Phys. 23, 327-341 (1977).

6 Berendsen, H. J., Postma, J. P. M., van Gunsteren, W. F., DiNola, A. & Haak, J. Molecular dynamics with coupling to an external bath. J. Chem. Phys. 81, 3684-3690, doi:10.1063/1.448118 (1984).
